# A cytokine receptor-masked IL2 prodrug selectively activates tumor-infiltrating lymphocytes for potent antitumor therapy

**DOI:** 10.1038/s41467-021-22980-w

**Published:** 2021-05-13

**Authors:** Eric J. Hsu, Xuezhi Cao, Benjamin Moon, Joonbeom Bae, Zhichen Sun, Zhida Liu, Yang-Xin Fu

**Affiliations:** 1 Department of Immunology, UT Southwestern Medical Center, Dallas, TX 75390 USA; 2 Department of Pathology, UT Southwestern Medical Center, Dallas, TX 75390 USA; 3 Department of Biomedical Engineering, UT Southwestern Medical Center, Dallas, TX 75390 USA

**Keywords:** Tumour immunology, Immunotherapy, Cancer

## Abstract

As a potent lymphocyte activator, interleukin-2 (IL-2) is an FDA-approved treatment for multiple metastatic cancers. However, its clinical use is limited by short half-life, low potency, and severe in vivo toxicity. Current IL-2 engineering strategies exhibit evidence of peripheral cytotoxicity. Here, we address these issues by engineering an IL-2 prodrug (ProIL2). We mask the activity of a CD8 T cell-preferential IL-2 mutein/Fc fusion protein with IL2 receptor beta linked to a tumor-associated protease substrate. ProIL2 restores activity after cleavage by tumor-associated enzymes, and preferentially activates inside tumors, where it expands antigen-specific CD8 T cells. This significantly reduces IL-2 toxicity and mortality without compromising antitumor efficacy. ProIL2 also overcomes resistance of cancers to immune checkpoint blockade. Lastly, neoadjuvant ProIL2 treatment can eliminate metastatic cancer through an abscopal effect. Taken together, our approach presents an effective tumor targeting therapy with reduced toxicity.

## Abstract (web summary)

While recognized as the first effective immunotherapy for human cancer, IL-2 based immunotherapy still encounters several limitations in the clinic. Here, the authors design a cytokine receptor-masked IL-2 mutein prodrug that is selectively activated by matrix metalloproteinases in the tumor microenvironment, promoting anti-tumor immune response while minimizing systemic toxicity.

## Introduction

Interleukin-2 (IL-2) is a potent cytokine that activates cytotoxic T cells and NK cells^1^. Because it so potently induces immune system activation, IL-2 was approved by the FDA as a treatment for renal cell carcinoma and metastatic melanoma^2^. Unfortunately, IL-2 cancer immunotherapy encounters three major issues in the clinic. First, IL-2 does not specifically activate cytotoxic lymphocytes (CTLs) in the tumor and is used up by many peripheral IL-2 sinks, such as Tregs and non-tumor-specific effector immune cells^3^. As a result, it has a short half-life^4^. Second, IL-2 binds with more affinity to the high-affinity IL-2 receptor alpha (IL2Rα)/beta/gamma complex, which is constitutively expressed on Tregs, than to the lower affinity IL-2 receptor beta (IL2Rβ)/gamma complex, which is expressed on CD8 T cells and NK cells^1,5–7^. Third, in part due to this high affinity for the trimeric high-affinity receptor complex on immunosuppressive Tregs, a higher dose of IL-2 is necessary to achieve CTL-mediated immune killing of the tumor, which leads to severe toxicity in most patients^2^.

Multiple strategies have been tested to address these three limitations. Synthesizing IL-2/Fc fusion proteins increases IL-2 half-life but can still induce major toxicity^8,9^. PEGylation of IL-2 can significantly improve IL-2 half-life but generation of consistent site-specific PEGylated IL-2 forms may be a challenge to manufacturers. Most PEGylated proteins are prepared through non-site-specific PEGylation, and thus equivalent number of PEGylations between batches may be difficult to achieve^10,11^. In addition, lack of tumor-targeted release of PEGylated IL-2 results in toxicity before reaching therapeutic doses, which limits repeatable results and significant objective responses in clinical trials^8,11,12^. Other groups have synthesized IL-2 muteins that either reduce IL-2 binding to IL2Rα or increase IL-2 binding to IL2Rβ on CD8 T cells^13–15^. Furthermore, biased IL-2/anti-IL2 antibody complexes preferentially bind to IL2Rβ on CD8 T cells as well^16–19^. Such variants can potentially induce decreased IL2Rα-mediated pulmonary toxicity, but still expand peripheral CTLs^16^. Depletion of CD8 T cells and presence of Tregs have been observed to decrease IL-2-mediated toxicity in humanized mouse systems^20^. Therefore, increased IL-2 selectivity of CD8 T cells may reduce its targeting and activation of Tregs but may also potentially increase overall toxicity. There is an urgent need to target IL-2 binding to effector cells specifically in tumor tissues without causing peripheral toxicity.

In this study, we synthesize a cytokine receptor-masked IL-2 prodrug (ProIL2) that exhibits minimal peripheral cytotoxicity. ProIL2 is cleaved and activated by proteases that are preferentially highly expressed in tumors into its free form, which is an IL-2 mutein denoted as SumIL2-Fc. The mutations on SumIL2 include “superkine” IL-2 mutations and the F42A mutation, which have both been previously described and tested to increase bias towards CD8 T cells^13,15^. ProIL2 preferentially expands CTLs in the tumor and has equivalent antitumor efficacy so SumIL2-Fc. Matrix metalloproteinases (MMPs) have been observed to play a role in tumor angiogenesis and invasion by digesting cell–matrix adhesions in cancer tissues, and are thus highly expressed on multiple tumor types^21^. Because they are highly expressed in many tumors and on inflammatory cells, we choose to use MMPs as our target tumor protease^22^. Overall, our effective and safe immunotherapy both presents a powerful option for combatting difficult clinical issues in the cancer field and serves as a platform for developing potent cytokine and antibody therapies in the future.

## Results

### Optimizing ProIL2 blocking, cleavage, and antitumor efficacy

**Fig. 1:**
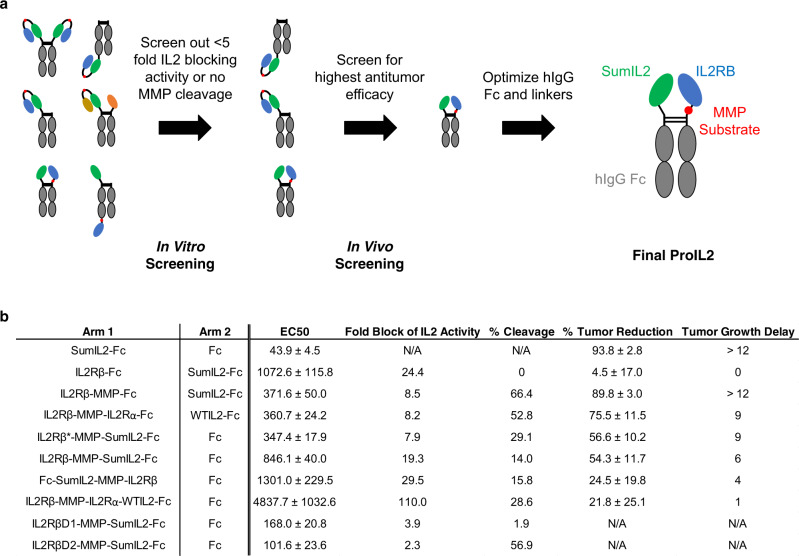
Engineering ProIL2 design. **a** Pipeline for designing and screening the most optimal ProIL2 designs. ProIL2 designs were screened using HEK-Blue^TM^ IL-2 reporter cell assay, MMP cleavage assay for 4 h, and tumor inoculation and growth measurement as described in the “Methods”. **b** Multiple designs of ProIL2 were tested. The Fc arms, listed from N terminus to C terminus in each entry, of each proposed design are shown. HEKBlue^TM^ IL-2 reporter cell assay was used to determine the EC50 in nM of functional activity of each ProIL2 design. Fold block of IL-2 activity was measured by dividing the EC50 of each ProIL2 design to that of SumIL2-Fc. To compare between experiments, SumIL2-Fc EC50 and construct EC50 were normalized to the displayed EC50 value. Each ProIL2 design was cleaved by MMPs and run on SDS-PAGE, where the amount of cleaved vs uncleaved ProIL2 product was quantified. MC38 s.c. tumor-bearing mice were injected i.p. with PBS and an equimolar dose of the listed ProIL2 design 9 days after tumor inoculation. 12 days after treatment, tumor volumes of PBS-treated and ProIL2-treated mice were measured, and the decrease in tumor volume in ProIL2-treated mice on this day was quantified. Tumor growth delay is defined as the difference in days it takes for treatment vs PBS groups to reach an average MC38 tumor volume of 150 mm^3^. Abbreviations: IL2Rβ = IL-2 receptor beta, IL2Rβ* = truncated version of IL-2 receptor beta, MMP = 10-mer cleavable MMP substrate, IL2Rα = IL2 receptor alpha, WTIL2 = wild-type IL-2, IL2RβD1 = IL-2 receptor beta domain 1, IL2RβD2 = IL-2 receptor beta domain 2. Data represent mean ± s.e.m. (*n* = 4 individual mice per group). Source data are provided as a Source Data file.

To allow IL-2 to preferentially target tumor-infiltrating lymphocytes (TILs) with long half-life, we designed ProIL2 to have four domains: human IgG1, an IL-2 variant, an IL-2 receptor, and a flexible MMP-cleavable linker. We modulate and engineer each of these four domains to maximize three criteria: blocking of IL-2 activity when uncleaved, MMP cleavability, and antitumor efficacy (Fig. 1a). We initially tested a homodimeric format of ProIL2, which, in N to C terminus order, incorporates IL2Rβ, the MMP-cleavable linker, and SumIL2 fused onto each “arm” of Fc. Intriguingly, this ProIL2 design exhibited no antitumor efficacy and could not be digested by MMPs, which raised the possibility that MMPs failed to access the cleavable linker (Supplementary Fig. 1a). As a result, we engineered designs of ProIL2 to minimize potential protein folding orientations that would result in interactions between IL2Rβ and Fc and prevention of MMP access of the cleavable linker. These engineering strategies involved synthesizing hIgG1 heterodimers^23^, incorporating different or truncated IL-2 receptor subunits, and altering the orientation of domains on both the N and C termini of each Fc arm. We initially rejected the designs that exhibited minimal IL-2 blocking activity and recombinant MMP cleavage. For each ProIL2 that passed in vitro screening, we injected MC38 tumor-bearing mice with phosphate buffered saline (PBS) or ProIL2 and quantified the amount of tumor reduction and tumor growth delay by each ProIL2 treatment (Fig. 1b). After determining which design exhibited the best antitumor efficacy, we tested modifications of the Fc region for this version of ProIL2. We observed that ProIL2 using wild-type hIgG1 Fc exhibited the same antitumor efficacy as using a mutant Fc that depletes antibody-dependent cell-mediated cytotoxicity^24^, but with reduced toxicity (Supplementary Fig. 1b–e). Thus, we chose to use wild-type hIgG1 Fc.

**Fig. 2:**
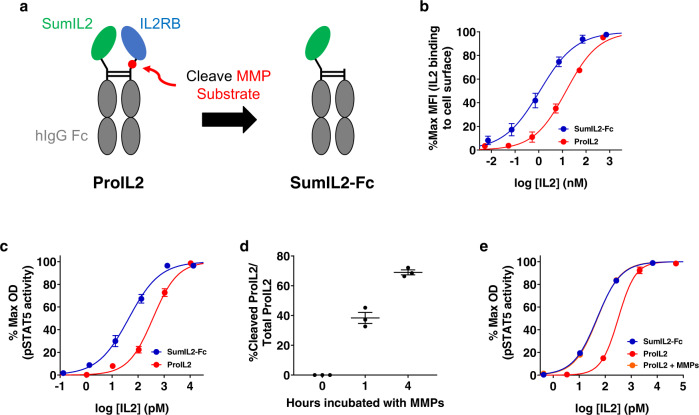
ProIL2 effectively masks IL-2 activity and is cleaved by MMPs in vitro. **a** Schematic of ProIL2 depicting that IL-2 receptor beta (IL2RB) binds and blocks IL-2 activity prior to cleavage (left) and activates into SumIL2-Fc upon MMP cleavage (right). **b** Binding of SumIL2-Fc and ProIL2 at different doses to HEK-Blue^TM^ IL-2 reporter cells was assessed via flow cytometry (*n* = 3 experiments, total 3 replicates per group). **c** Functional activity of SumIL2-Fc and ProIL2 was assessed by using HEK-Blue^TM^ IL-2 reporter cell assay (*n* = 3 experiments, total 9 replicates per group). **d** Protease activation of ProIL2. ProIL2 was incubated with human MMP2, MMP9, and MMP14 for 1 or 4 h, then run on non-reducing SDS-PAGE, where gel band intensity was subsequently quantified (*n* = 3 experiments). **e** Functional activity of SumIL2-Fc, ProIL2, and ProIL2 that was incubated with human MMP2, MMP9, and MMP14 for 4 h was assessed by using HEK-BlueTM IL-2 reporter cell assay (*n* = 1 representative experiment of 2 experiments, 3 replicates each group per experiment, total 6 replicates). Data represent mean ± s.e.m. Source data are provided as a Source Data file.

We ultimately determined that the design in Fig. 2a maximized our three screening criteria. On one Fc arm, we fused SumIL2 to the N terminus of wild-type Fc, linked together by a GGGGS linker. On the other Fc arm, we fused the full extracellular domain of IL2Rβ to the N terminus of Fc, linked together by a flexible linker that includes a synthetically determined 10-amino-acid MMP protease substrate (Fig. 2a and Supplementary Table 1). We purified and eluted this ProIL2 using Protein A chromatography and assessed its purity via SDS-PAGE, noting that we indeed created one single band of appropriate approximate size (Supplementary Fig. 2a). Purification of this protein was high yield. This protein is also very stable, not degrading and still having blocking/cleavage activity even after 4 months of storage at 4 °C (Supplementary Fig. 2b). Overall, we tested numerous designs of ProIL2, modifying each domain of ProIL2 until we synthesized the most optimal form of ProIL2. From here on, the phrase “ProIL2” refers to our most optimal prodrug design in Fig. 2a.

### ProIL2 blocks IL-2 activity when uncleaved

We tested the ability of IL2Rβ to block both SumIL2 binding and functional activity when ProIL2 was uncleaved. First, we used flow cytometry to stain for ProIL2 binding to the surface of cells that express IL-2 receptor subunits. Compared to SumIL2-Fc (EC_50_ = 1.1 ± 0.2 nM), ProIL2 (EC_50_ = 14.4 ± 1.4 nM) bound with 13-fold lower affinity to these cells (Fig. 2b and Supplementary Fig. 2c). We also used an HEK-Blue^TM^ IL-2 reporter cell assay to compare functional activity of these two proteins. Similarly to the binding assay, ProIL2 (EC_50_ = 371.6 ± 50.0 pM) also induced 8.5-fold less activity than SumIL2-Fc (EC_50_ = 43.9 ± 4.5 pM) (Fig. 2c and Supplementary Fig. 2d). Furthermore, the activity of ProIL2 is also less than that wild-type IL-2 linked to hIgG (WTIL2-Fc) or recombinant WTIL2, which both have activity comparable to that of SumIL2-Fc (Supplementary Fig. 2c, d).

We also assessed whether ProIL2 could cleave efficiently in vitro. We incubated ProIL2 with recombinant MMPs for multiple time points and then quantified the amounts of uncleaved and cleaved ProIL2 via SDS-PAGE (Supplementary Fig. 2a). MMPs are able to cleave ProIL2 gradually over time, cleaving 70% of the initial amount of ProIL2 over 4 h of incubation (Fig. 2d and Supplementary Fig. 2e). ProIL2 that has been sufficiently cleaved also has activity close to SumIL2-Fc, as demonstrated by IL-2 reporter cell assay (Fig. 2e). Taken together, our design of ProIL2 effectively blocks SumIL2-Fc binding and stimulatory activity and is avidly cleaved by MMPs in vitro.

### ProIL2 preferentially localizes to and is cleaved in tumors

**Fig. 3:**
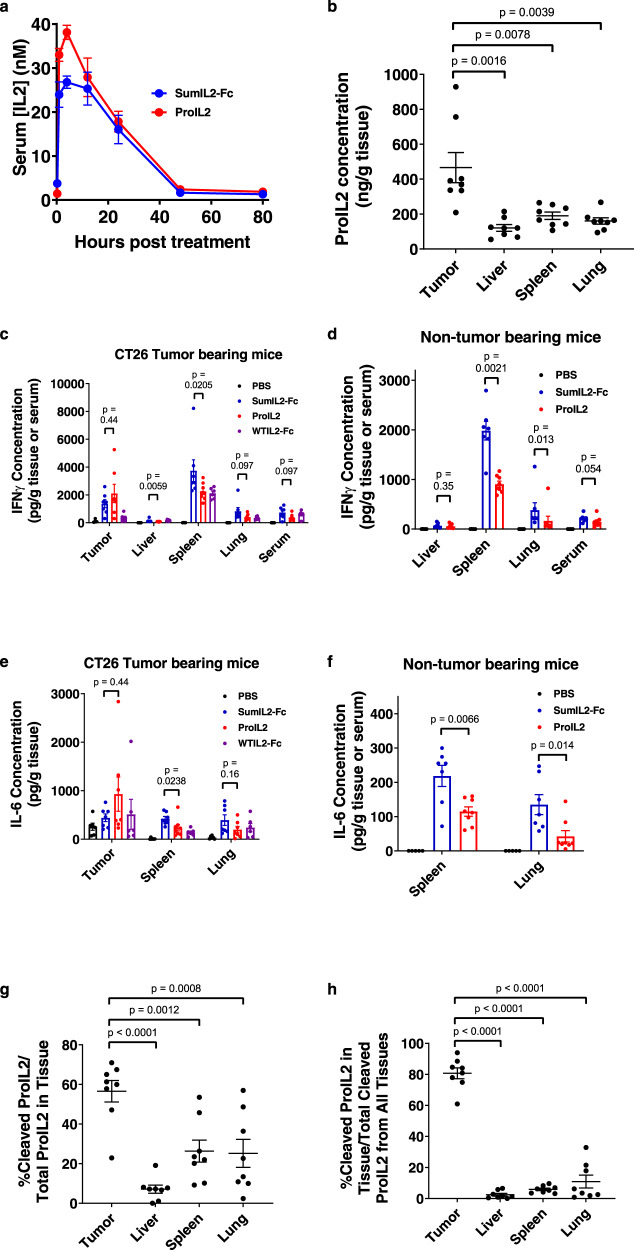
ProIL2 preferentially localizes to and cleaves effectively in tumors. **a** Equimolar doses of SumIL2-Fc or ProIL2 (300 pmol) was injected i.p. into CT26 s.c. tumor-bearing mice, and serum was collected and isolated at multiple time points. hIgG ELISA was used to quantify the amount of SumIL2-Fc or ProIL2 in the serum (*n* = 2 experiments, total 8 individual mice). **b**–**h** PBS or 200 pmol of SumIL2-Fc, ProIL2, or WTIL2-Fc was injected i.p. into CT26 s.c. tumor- or non-tumor-bearing mice, and the labeled tissues were collected and homogenized 24 h after treatment (*n* = 2 experiments, total 5 individual mice for PBS, 7 for SumIL2-Fc, 8 for ProIL2, 6 for WTIL2-Fc). **b** hIgG ELISA was used to quantify the amount ProIL2 in each homogenate, normalized by total tissue weight. **c**–**f** Cytometric Bead Array was used to quantify the amount of IFN or IL-6 in each tissue homogenate or serum. **g**, **h** hIgG immunoprecipitation of equivalent weights of tissue homogenate was performed with Protein A-binding beads, and then run on western blot with hIgG-binding antibody (*n* = 2 experiments, total 8 individual mice). **g** Relative gel band intensity per lane was quantified. **h** From each of the four tissues in one mouse, cleaved ProIL2 western blot bands were quantified relative to each other (i.e., the sum of cleaved ProIL2 bands from each of the four tissues = 100%). Data represent mean ± s.e.m. Mann-Whitney *U* tests were performed to calculate *p* values. Source data are provided as a Source Data file.

Because ProIL2 cleaved well in response to MMPs, we assessed whether ProIL2 could also be cleaved in vivo, preferentially in the tumor. We first quantified the serum half-life of ProIL2. After injecting ProIL2 intraperitoneally into tumor-bearing mice, we calculated its serum half-life to be 15.1 h (Fig. 3a). This is much longer than that of recombinant IL-2 (<15 min)^25,26^ and comparable to the 14.6 h of SumIL2-Fc.

To determine how ProIL2 distributed into and cleaved in different tissues, we injected ProIL2 into CT26 tumor-bearing BALB/c mice and extracted and homogenized tumors, livers, spleens, and lungs 24 h after injection. We first quantified the amount of ProIL2 in each tissue homogenate. Two to four times more ProIL2 per total amount of tissue localized into tumors compared to all of the other tissues (Fig. 3b and Supplementary Fig. 3a). We also determined the levels of multiple inflammatory cytokines in each tissue in response to ProIL2. Tumor tissue showed a high amount of IFNγ, IL-6, and TNFα (Fig. 3c–f and Supplementary Fig. 3b), which is consistent with a phenotype of TIL activation^27–29^. Compared to SumIL2-Fc, we also observed that ProIL2 induced similar amounts of cytokines in the tumor and fewer peripheral inflammatory cytokines in the periphery. This also held true in non-tumor-bearing mice, where compared to CT26-bearing mice, the total levels of peripheral cytokines were halved but ProIL2 still induced fewer cytokines than did SumIL2-Fc (Fig. 3c–f and Supplementary Fig. 3c).

We also aimed to quantify whether and how much ProIL2 would cleave in tumors in vivo. We used Protein A immunoprecipitation and western blot analysis to quantify the amount and ratio of uncleaved and cleaved ProIL2 in each extracted tissue homogenate (Supplementary Fig. 3d). On average, over 50% of ProIL2 in the tumor was cleaved, compared to less than 25% in the liver, lung, and spleen (Fig. 3g). A similar amount of cleavage in these non-tumor tissues was also observed in non-tumor-bearing mice (Supplementary Fig. 3e). We also compared the amount of cleaved ProIL2 between tissues. In each set of tissues from one mouse, we quantified the amounts of cleaved ProIL2 in each tissue relative to each other, with the total amounting to 100% of cleaved ProIL2 in the whole mouse. We observed that cleaved ProIL2 product was most prevalent in the tumor compared to any other tissue (Fig. 3h). Similar results were observed in a B16 tumor-bearing mouse model (Supplementary Fig. 4). Altogether, these results demonstrate that ProIL2 preferentially localizes to and cleaves in tumors but not in non-tumor tissues.

### ProIL2 reduces toxicity without reducing antitumor efficacy

**Fig. 4:**
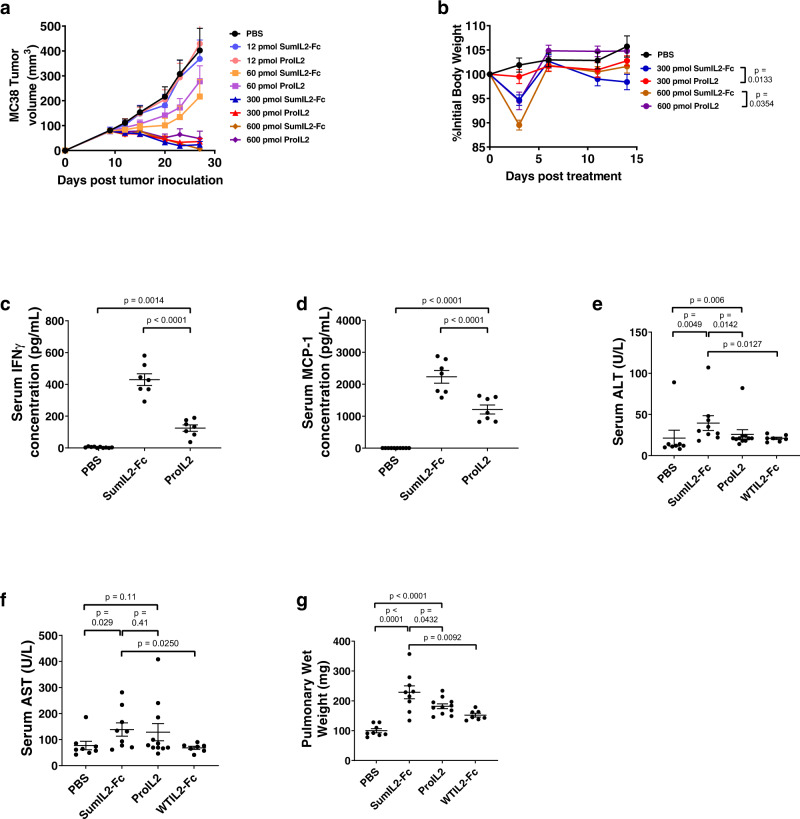
In vivo antitumor efficacy and toxicity assessment of ProIL2. **a**, **b** MC38 s.c. tumor-bearing mice were injected i.p. with one dose of the labeled treatment on day 9 post tumor inoculation; tumor growth and body weight were measured (*n* = 2 experiments, total 6 individual mice for 12 pmol treatment groups, 7 for 600 pmol treatment groups, 8 for 60 and 300 pmol treatment groups). **c**, **d** B16 s.c. tumor-bearing mice were injected i.p. with PBS, SumIL2-Fc or ProIL2 (120 pmol) one time on day 9 post tumor inoculation. Serum was collected and isolated from mice 24 h post treatment, and Cytometric Bead Array was used to quantify the amount of serum IFN or MCP-1 (*n* = 2 experiments, total 10 individual mice for PBS treatment group, 7 for other groups). **e**–**g** B16 s.c. tumor-bearing mice were injected i.p. with PBS, SumIL2-Fc, ProIL2, or WTIL2-Fc (150 pmol) two times on days 9 and 12 post tumor inoculation. Serum and lungs were collected 48 h after the last treatment. Serum ALT and AST were quantified, along with pulmonary wet weight as described in the “Methods” (*n* = 3 experiments, total 8 individual mice for PBS treatment group, 9 for SumIL2-Fc, 11 for ProIL2, 7 for WTIL2-Fc). Data represent mean ± s.e.m. Student’s *t* tests were performed to calculate *p* values. Source data are provided as a Source Data file.

We have emphasized the necessity of reducing peripheral toxicity with our prodrug. However, its antitumor efficacy and ability to expand TILs should be comparable to that of SumIL2-Fc. We intraperitoneally injected equimolar doses of ProIL2 or SumIL2-Fc into MC38 tumor-bearing mice and observed that while ProIL2 induced significantly less body weight loss compared to SumIL2-Fc, the two treatments exhibited equivalent efficacy in causing tumor regression (Fig. 4a, b and Supplementary Figs. 5 and 6a). At our lowest observed dose of maximum antitumor efficacy, we consistently observed this phenomenon in multiple tumor models (Supplementary Fig. 6b–e).

We also examined cytokine-related toxicity of ProIL2. We treated tumor-bearing mice with PBS, SumIL2-Fc, or ProIL2, collected serum from these mice 24 h post treatment, and quantified the level of serum inflammatory cytokines. We observed that compared to SumIL2-Fc, ProIL2 exhibits markedly reduced serum levels of inflammatory markers IFNγ and MCP-1 (Fig. 4c, d). We also collected sera, livers, and lungs from tumor-bearing mice treated with PBS, SumIL2-Fc, ProIL2, or WTIL2-Fc. Compared to SumIL2-Fc treatment, ProIL2 induced less increases in serum AST and ALT levels, reduced amounts of pulmonary edema, and decreased lymphocytic infiltration into livers (Fig. 4e–g and Supplementary Fig. 6f). In all of these cases, WTIL2-Fc induced less toxicity compared to SumIL2-Fc-treated mice. Furthermore, while we observed the presence of mouse-produced serum antibodies against SumIL2-Fc, these antibodies appeared to target hIgG rather than IL-2 (Supplementary Fig. 6g). Overall, ProIL2 induces decreased off-target toxicity in non-tumor tissues compared to SumIL2-Fc and therefore provides a wider therapeutic window than SumIL2-Fc.

**Fig. 5:**
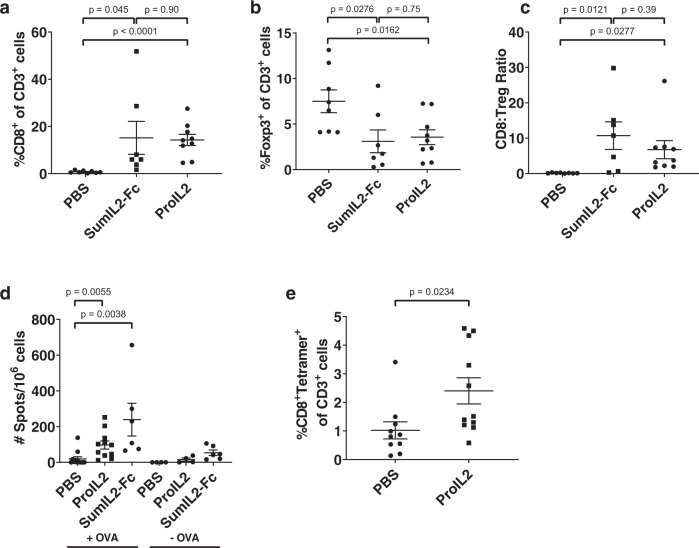
In vivo flow cytometry analysis of ProIL2. **a**–**c** B16 s.c. tumor-bearing mice were injected i.p. with PBS, SumIL2-Fc or ProIL2 (300 pmol) two times on days 9 and 12 post tumor inoculation. 48 h after the last treatment, mice were euthanized and tumors were extracted, digested in collagenase/DNAse, and resuspended as single cells. Flow cytometry was used to quantify the amount of CD8 T cells, amount of Tregs, and CD8:Treg ratio in each respective group (*n* = 2 experiments, total 8 individual mice for PBS treatment group, 7 for SumIL2-Fc, 9 for ProIL2). **d** B16-OVA s.c. tumor-bearing mice were injected i.p. with PBS or ProIL2 (300 pmol) one time on day 9 post tumor inoculation. 5 days after treatment, draining lymph nodes were collected, IFNγ ELISPOT analysis was performed and spots were quantified (*n* = 2 experiments, total 12 individual mice for PBS/OVA group, 11 for ProIL2/OVA, 6 for SumIL2-Fc/OVA, 4 for PBS/no OVA, 4 for ProIL2/no OVA, 6 for SumIL2-Fc/no OVA). **e** B16-OVA s.c. tumor-bearing mice were injected i.p. with PBS or ProIL2 (300 pmol) one time on day 9 post tumor inoculation. 5 days after treatment, mice were euthanized and tumors were extracted, digested in collagenase/DNAse, and resuspended as single cells. Flow cytometry was used to quantify the amount of OVA-tetramer CD8 T cells (*n* = 3 experiments, total 10 individual mice for PBS treatment group, 11 for ProIL2). Data represent mean ± s.e.m. Student’s *t* tests were performed to calculate *p* values. Source data are provided as a Source Data file.

While ProIL2 reduces immune activation outside of the tumor, we still want to ensure that ProIL2 potently activates intratumor CTLs. CD8 T cells, Tregs, and dendritic cells have been observed to be involved in antitumor efficacy, so we focused on these particular immune-cell populations^30^. We extracted and resuspended tumors and spleens into single suspensions from PBS-, SumIL2-Fc-, or ProIL2-treated tumor-bearing mice 72 h after treatment. We then used flow cytometry to quantify immune-cell levels in the tumor (Supplementary Fig. 7a–d) and spleens. We observed that ProIL2 expands a lower percentage of CD8 T cells and dendritic cells in the spleen relative to SumIL2-Fc, which further supports its decreased potential for toxicity (Supplementary Fig. 7e–h). We demonstrate that both SumIL2-Fc and ProIL2 increased CD8 T cells in the tumor without increasing Tregs, thus inducing a high CD8:Treg ratio (Fig. 5a–c and Supplementary Fig. 7i, j). More specifically, using a B16-OVA tumor model, we observe an increase in OVA-antigen-specific CD8 T cells in response to ProIL2 treatment (Fig. 5d, e and Supplementary Fig. 7l). In the enzyme-linked immunosorbent spot (ELISPOT) experiment, small amounts of IFNγ spots in non-OVA peptide-treated lymphocytes may be attributed to nonspecific activation of CTLs in the periphery that ultimately circulate to the tumor and thus tumor-draining lymph nodes. These results suggest that ProIL2 induces a strong activating phenotype in the tumor microenvironment (TME), particularly a tumor-antigen-specific phenotype. However, ProIL2 and SumIL2-Fc both decreased the frequency of dendritic cells in the tumor (Supplementary Fig. 7k). These in vivo experiments demonstrate that ProIL2 activates tumor-specific immunity in the TME and minimally stimulates toxic cytokine inflammatory responses in the periphery.

### ProIL2 can overcome resistance to immune checkpoint blockade (ICB)

**Fig. 6:**
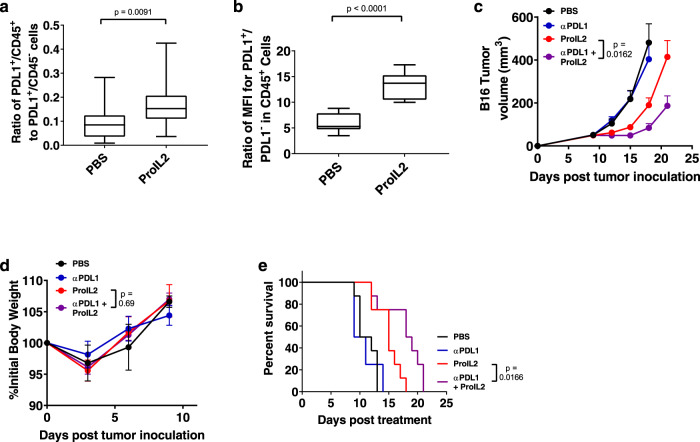
ProIL2 can overcome tumor resistance to ICB. **a**, **b** B16 s.c. tumor-bearing mice were injected i.p. with PBS or ProIL2 (300 pmol) one time on day 9 post tumor inoculation. 72 h after treatment, mice were euthanized and tumors were extracted, digested in collagenase/DNAse, and resuspended as single cells. Flow cytometry was used to quantify the amount of PDL1+ and CD45+ cells. In CD45+ cells, the MFI of PDL1+ and PDL1- cells were compared. Box and whisker plots with maxima, 75th percentile, center, 25th percentile, and minima are displayed (*n* = 3 experiments, total 12 individual mice per group). **c**, **d** B16 s.c. tumor-bearing mice were injected i.p. with PBS, anti-PDL1 (200 μg each), ProIL2 (300 pmol), or anti-PDL1 and ProIL2 one time on day 9 post tumor inoculation; tumor growth and body weight were measured (*n* = 3 experiments, total 12 individual mice for PBS and ProIL2 treatment groups, 13 for anti-PDL1 and combination groups). **e** Mice were analyzed for survival starting the day after treatment. For mouse health, mice were euthanized when tumors reached a volume of greater than 1000 mm^3^ (*n* = 2 experiments, total 8 individual mice per group). Data represent mean ± s.e.m. Student’s *t* tests (**a**–**d**) or log-rank tests (**e**) were performed to calculate *p* values. Source data are provided as a Source Data file.

ProIL2 may activate lymphocytes to produce IFN or other cytokines, thus upregulating PD-L1 on both host and tumor cells. We aimed to expose whether host or tumor cells respond to IL-2-mediated cytokines for PD-L1 expression. We observed that ProIL2 significantly increases the PDL1^+^ CD45^+^/CD45^−^ ratio (Fig. 6a and Supplementary Fig. 8a, b). We hypothesize that this particular expansion of cell populations would maximize the binding of anti-PDL1 to the PDL1^+^ host cells that are required for antitumor efficacy of PDL1 blockade^31,32^. We also observe that in CD45^+^ populations, the MFI level of PDL1 increases more in ProIL2-treated tumors than in PBS-treated tumors (Fig. 6b and Supplementary Fig. 8c–e). As a result, both the increased frequency and increased expression of PDL1 in host cells in the TME suggest that ProIL2 therapy may sufficiently stimulate immune cells in the TME for synergy with anti-PDL1 treatment.

We inoculated mice with B16 tumors and treated these mice with PBS, anti-PDL1, ProIL2, or ProIL2/anti-PDL1. These tumors were resistant to either anti-PDL1 or ProIL2 single therapy, as neither treatment could significantly control these tumors. However, the combination of ProIL2 and anti-PDL1 controlled these established tumors much more effectively without causing body weight loss (Fig. 6c, d and Supplementary Fig. 8f–i). This combination also increases the survival length of treated mice compared to any other treatment group (Fig. 6e). These data suggest that ProIL2 can effectively overcome resistance to ICB in unresponsive tumors, still without significant toxicity.

### Preoperative ProIL2 treatment eliminates metastatic disease

Surgical removal of tumors has remained one of the most effective treatments of primary cancers. However, surgery is not able to cure metastases, and the resulting surgical wound inflammation may potentially even be a trigger for the formation of metastatic disease^33^. In order to prevent postoperative metastatic disease, many groups have proposed using neoadjuvant immunotherapy to generate a number of T cells that may be sufficient to patrol and outnumber metastatic tumor cells.

**Fig. 7:**
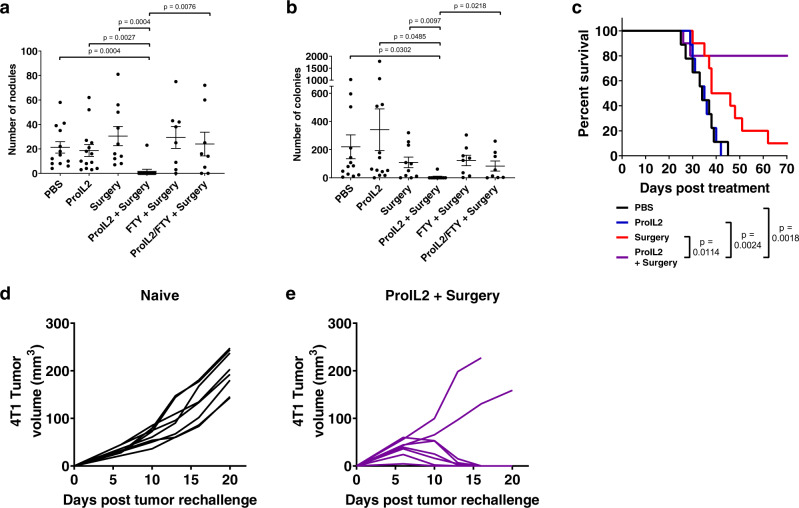
Preoperative ProIL2 combined with surgery can eliminate metastases through an abscopal effect. 4T1 s.c. tumor-bearing mice were injected i.p. with PBS or ProIL2 (300 pmol) on days 9 and 12 post tumor inoculation. Mice treated with FTY720 were i.p. treated with 10 μg every other day for 14 days starting on day 8 post tumor inoculation. On day 14 post tumor inoculation, the listed mice had their tumors surgically removed. **a** 25 days post initial treatment, mice were euthanized and metastatic nodules in the thoracic cavity were counted. **b** 25 days post initial treatment, mice were euthanized and lungs were extracted, digested in collagenase/DNAse, and resuspended as single cells. Resuspended cells were plated at a 1:400 ratio with 6-thioguanine selection medium, and the number of colonies were counted 6 days post seeding (*n* = 3 experiments, total 13 individual mice for PBS treatment group, 10 for Surgery, 14 for ProIL2 and ProIL2/Surgery, 8 for FTY/Surgery and FTY/ProIL2/Surgery). **c** Mice were analyzed for survival starting the day after the first i.p. injection treatment (*n* = 2 experiments, total 9 individual mice for PBS and ProIL2 treatment groups, 10 for Surgery and ProIL2/Surgery). Data represent mean ± s.e.m. Student’s *t* tests (**a**, **b**) and log-rank tests (**c**) were performed to calculate *p* values. **d**, **e** Either naive BALB/c mice or ProIL2/surgery cured mice were rechallenged with s.c. 4T1 tumors in the opposite flank of primary tumor injection. Tumor growth was measured and displayed as individual curves (*n* = 2 experiments, total 8 individual mice per group). Source data are provided as a Source Data file.

We subcutaneously inoculated mice with 4T1 mammary tumors, which spontaneously metastasize to the lung. We preoperatively treated these tumor-bearing mice with either PBS or ProIL2 and resected tumors from half of these mice. Approximately 3 weeks after tumor resection, we extracted lungs from these mice and quantified the number of metastatic nodules and colonies in each lung. We observed that while any single treatment group had many metastatic nodules and colonies, almost no mice in our preoperative ProIL2 and surgery group had any nodules or colonies (Fig. 7a, b). We observed that 80% of preoperative ProIL2 and surgery-treated mice survived, whereas the majority of mice in any other treatment group died within the time course of this study (Fig. 7c). We also rechallenged these surviving ProIL2/surgery-treated mice with 4T1 tumors at a secondary location and observed that 75% of these mice exhibited no tumor relapse (Fig. 7d, e). These studies demonstrate that preoperative ProIL2 and surgery treatment is vastly superior to ProIL2 or surgery alone in eradicating metastases and can also prevent cancer recurrence.

We subsequently sought to determine how ProIL2 can prevent metastases. One potential model is that ProIL2 can flow into lung tissues and activate residual lymphocytes in the lung to control metastatic disease. However, we hypothesized that TILs activated by ProIL2 are necessary for the prevention of metastases. To investigate whether preoperative ProIL2 could generate T cells that migrate into distal sites, we additionally administered FTY720, which binds to sphingosine-1-phosphate receptors in lymph nodes to prevent lymphocyte egress, to preoperative control and ProIL2-treated mice. The majority of lymphocytes traffic from organs into draining lymph nodes. Because FTY720 prevents lymphocytes from leaving these draining lymph nodes, FTY720 essentially prevents lymphocytes in different tissues (i.e., tumor, tumor-draining lymph nodes) from trafficking into other tissues (i.e., lung). We observed that while combined ProIL2 and surgery-treated mice prevented metastatic nodule and colony growth, addition of FTY720 to combined ProIL2 and surgery treatment abrogated this effect (Fig. 7a, b). As FTY720 prevents lymphocytes outside the lung from entering into the lung, these data suggest that residential lymphocytes in the lung are insufficient for metastatic control, and thus require TILs. These results suggest that neoadjuvant ProIL2 induces an abscopal effect that is effective and required for eradicating metastases.

## Discussion

IL-2 was initially considered to be a promising potent antitumor therapy. However, its low half-life, low tolerability, and induction of cytokine release syndrome significantly limits its use in most human patients. We engineered and generated a unique IL-2 prodrug that combines a potent CD8 T cell-preferential IL-2 mutein with a blocking IL-2 receptor that can be released by tumor-associated MMPs. This next-generation pro-cytokine incorporates dual advantages of having potent immunostimulatory activity in the tumor and side effect minimization through tumor-specific targeting. Furthermore, our ProIL2 can effectively synergize with current standard of care cancer therapies to overcome ICB-resistant and even metastatic cancers.

We proposed the concept of an IL-2 prodrug in order to address limitations of not only recombinant IL-2, but also of current next-generation IL-2 designs. One popular IL-2 variant (PEG-IL2) involves attaching PEG to regions of IL-2 that normally bind to IL-2 receptors, thus decreasing peripheral activity of IL-2^11^. However, production of consistently PEGylated regions of IL-2 is not easy, and PEG may nonspecifically detach from IL-2^10,11^. In contrast to PEG-IL2, for which PEGylation and additional conjugation processes may produce mixed products, ProIL2 can be produced at a high yield as a single molecule very quickly and efficiently. Therefore, synthesis of ProIL2 would comparatively ease any concerns manufacturers may have about the production and mixed nature of PEG-IL2. ProIL2 is also significantly preferentially active in the tumor.

A second IL-2 design fuses IL-2 to an antibody, such as epidermal growth factor receptor, which “actively” targets specific proteins on tumor cells. However, upon binding of antibodies to antigens on the tumor surface, bound proteins are endocytosed, in this case potentially before IL-2 can stimulate lymphocytes^34^. Also, even when paired with antibodies, such high-affinity IL-2 variants will still target and activate T cells in the periphery. On the other hand, ProIL2 “passively” targets MMPs that are secreted by tumor cells or are attached to inflammatory cells in the TME, thus decreasing the concern of endocytosis prior to IL-2 activation of CTLs. Furthermore, specifically targeted antibody/IL-2 fusions require tumors to specifically express the target antigen. Because so many tumors express MMPs, ProIL2 is not as limited in terms of the expression of a targeted tumor antigen.

Other considerable IL-2 engineering designs include strategies to increase binding to CD8 T cells or decrease binding to IL2Rα on Tregs. The “superkine” IL-2 is one of the most notable IL-2 muteins synthesized for this purpose, and these mutations are included in SumIL2^13^. Superkine IL-2 also shows evidence of reduced pulmonary edema compared to WT-IL2. However, in all of our toxicity experiments (cytokines, lung, and liver toxicity), SumIL2-Fc still exhibited an increased toxicity profile compared to WTIL2-Fc, thus suggesting an increase in both antitumor efficacy and toxicity with these mutations. Another design complexes IL-2 to an anti-IL2 antibody to decrease binding to IL2Rα^16–19^. Both superkine IL-2 and this IL-2 antibody complex significantly increase splenic CD8 T cell expansion compared to WT-IL2. Despite reduced evidence of pulmonary toxicity in these mouse models, this CD8 T cell expansion may still potentially result in cytotoxicity in human patients, as humanized mouse models demonstrate that IL-2 toxicity is dependent on CD8 T cells^20^. One potential limitation with this humanized mouse model is that there is no IL-2 receptor gamma on non-immune cells to receive any sort of IL-2 signaling. It has previously been identified that IL-2 can induce pulmonary edema through IL2Rα on pulmonary non-immune cells in immune-depleted murine tumor models^16^. It is thus unclear how much balance lies between direct effects of IL-2 on pulmonary toxicity and peripheral CTL-mediated effects, and these mechanisms of toxicity must be evaluated further. Still, in light of observations of even some potential toxicity from these constructs, we still deem a significant need for ProIL2, which reduces both tissue toxicity and splenic CD8 T cell expansion compared to the potent SumIL2-Fc but still maintains equivalent antitumor efficacy.

A protease-activated IL-2 fusion protein was designed to attempt to block wild-type IL-2 (WT-IL2) with IL2Rα^35^. However, this fusion does not contain any modality to extend half-life, such as Fc. Furthermore, this fusion uses WT-IL2 and thus has higher affinity for Tregs when cleaved. To reduce the preferential targeting of IL-2 to Tregs, we designed IL-2 variants with mutations that lower IL2Rα-binding affinity. IL2Rα failed to block this mutant IL-2, likely activating CTLs before reaching tumor tissues. Moreover, a design consisting solely of WT-IL2 and IL2Rα appears to strongly dimerize with other WT-IL2/IL2Rα complexes, thus potentially decreasing access to the protease substrate linker due to interaction of different domains of this fusion protein^36^. ProIL2 with Fc has a long half-life, high affinity for CD8 T cells, and includes an Fc domain that minimizes potential dimerization. Overall, while many other next-generation IL-2 designs have been tested, they encounter limitations that we address with ProIL2.

MMP expression has also been observed to increase as tumors become more invasive or metastatic, and is expressed in a wide range of cancers^22,37–40^. Therefore, we chose to use MMPs based on its specificity and abundance in cancers. Our lab then used mutational screening to synthesize a 10-amino-acid peptide substrate that would most effectively cleave in the presence of murine and human tumor fragments. As observed, significant levels of ProIL2 are cleaved in response to MMPs within 1 h in vitro and inside tumors within 24 h in vivo (Figs. 2d and 3d). This suggests that ProIL2 can favorably become an active cytokine in the tumor quite shortly after injection. Strikingly, very little cleaved ProIL2 was in the liver. The liver can express many proteases such as cathepsins, plasminogen activators, and metalloproteases^41–43^. The liver is heavily involved in clearance of different proteins in the host, and proteinases may potentially ultimately end up in the liver. The lack of cleaved ProIL2 in the liver suggests that our MMP substrate is minimally affected by these hepatic proteases and is thus more specific to MMP cleavage.

Despite this clear reduction in toxicity, we can still observe some evidence of peripheral toxicity. We observe evidence of a small amount of peripheral cleavage, tissue inflammatory cytokine release, and lung and liver toxicity in response to ProIL2 treatment (Fig. 4 and Supplementary Figs. 3, 4, and 6). There is evidence of this in non-tumor-bearing mice as well. Baseline levels of MMPs in the various peripheral tissues, i.e., MMPs from neutrophils and macrophages^44,45^, may be causing this cleavage and toxicity. The immunogenicity caused by this human-based protein in mouse model may result in additional myeloid cell responses, and because MMPs are secreted more by activated myeloid cells, may result in increased cleavage of ProIL2^44–46^. Because these antibodies are specific to human IgG1 on ProIL2, it is possible that this cleavage and immunogenicity will be decreased in human patients. A ProIL2/antibody fusion, especially with antibodies targeting IL-2 to TILs, could potentially decrease off-target cleavage of ProIL2.

Despite the “release of brakes” by ICB, only a small fraction of patients respond to ICB. For anti-PDL1 in particular, we propose that these patients exhibit resistance to anti-PDL1 for two major reasons. First, some patients may have lower expression of PDL1 in the TME^47,48^. Second, even after anti-PDL1 treatment, limited T cell activating and expanding cytokines in the TME may prevent effector lymphocytes from being fully stimulated. Therefore, T cell growth and activating cytokines are required for rapid and effective T cell activation and expansion, especially after anti-PDL1 treatment. We have observed that ProIL2 can “fuel” immune cells and overcome their dysfunctional state. In particular, anti-PDL1 treatment is dependent on host cell PDL1 expression^49,50^, and ProIL2 is able to both expand the number of host PDL1^+^ cells and increase the expression level of PDL1 on host cells (Fig. 6a, b). Furthermore, after anti-PDL1 treatment, ProIL2 also provides the follow-up stimulation to antigen-specific CD8 T cells in the TME to induce tumor regression (Figs. 5 and 6c). This results in decreased mortality in combination therapy-treated mice (Fig. 6e). Therefore, ProIL2 overcomes anti-PDL1-resistant tumors, thus providing a safe option for synergistic, powerful combination therapy against tumors that may be difficult to treat.

Tumor resection is an effective modality for curing patients of primary tumors. However, most of these patients still relapse from metastasis. Immunotherapy can potently prime TILs to egress from tumors and combat metastatic cancer cells, but can often fail to significantly overcome large tumor burdens, especially those that continue to metastasize and disseminate into peripheral tissue^51^. Therefore, the hypothesis that combining neoadjuvant immunotherapy, which would activate and egress a sufficient number of tumor-specific T cells, with surgery can eradicate a relatively small number of metastatic tumors at distal sites has been proposed. Neoadjuvant immunotherapy, including preoperative IL-2, has improved patient responses to surgery, likely due to improved tumor-specific CTL expansion^52–54^. We also proposed that ProIL2, as a potent tumor targeting immunotherapy, would effectively activate TILs which would be essential for the control of metastatic disease. We initially demonstrate that neoadjuvant ProIL2 can indeed eliminate metastatic cancer very effectively (Fig. 7). However, when we also inhibited intratumor CTLs from leaving the tumor with FTY720, we observed that ProIL2 could no longer control distal lung metastases (Fig. 7a, b). These results are consistent with our hypothesis that neoadjuvant immunotherapy can prime and activate tumor-specific CTLs which can exit the tumor to eradicate distant metastases.

Taken together, our work has designed and synthesized an immunocytokine therapy that is safe but still exhibits potent antitumor efficacy. In particular, ProIL2 preferentially targets effector TILs without substantially inducing Tregs or peripheral lymphocytes, thus overcoming a large hurdle for IL-2 therapy. Administration of ProIL2 also overcomes advanced and metastatic tumor resistance to current standard of care therapies. Overall, our study has provided a low toxicity but effective immunotherapy that can overcome clinically relevant advanced cancers and metastasis.

## Methods

### Mice

Female C57BL/6J and BALB/c mice were purchased from The Jackson Laboratory. All mice were maintained under specific pathogen-free conditions, at 72 °F and 30–70% relative humidity, and under a 12 h dark/12 h light cycle. Animal care and experiments were carried out under institutional and National Institutes of Health protocol and guidelines. This study has been approved by the Institutional Animal Care and Use Committee of the University of Texas Southwestern Medical Center.

### Cell lines and reagents

B16, MC38, CT26, and 4T1 cell lines were purchased from American Type Culture Collection (Supplementary Table 2). Cell lines were authenticated by morphology, karyotyping, and PCR testing. All cell lines were routinely tested using mycoplasma contamination kit (R&D) and cultured in Dulbecco’s modified Eagle’s medium supplemented with 10% heat-inactivated fetal bovine serum, 100 U/mL penicillin, and 100 U/mL streptomycin under 5% CO_2_ at 37 °C. Anti-PDL1 (Atezolizumab) mAb was purchased from Genentech. FTY720 was purchased from Selleckchem.

### Flow cytometry analysis

Unless specifically listed otherwise, all flow cytometry antibodies were stained at a 1:100 dilution. Single cell suspensions from either spleen, tumor, or in vitro co-cultured cells were incubated with anti-FcγIII/II receptor (clone 2.4G2) for 15 min to block nonspecific binding before staining with the conjugated antibodies. To exclude dead cells, 1:1000 Fixable Viability Dye eFluor^TM^ 506 was used. Foxp3 was stained intracellularly by using True-Nuclear transcription factor buffer set (BioLegend) following the manufacturer’s instructions.

Example gating schemes for major flow cytometry experiments are displayed in Supplementary Fig. 7a–d. For all flow cytometry experiments, cells were first sorted by size (FSC/SSC and FSC-height/width), and then viability (eFluor^TM^ 506 Viability Dye). For gating CD8 T cells and Tregs, CD45^+^/CD3^+^ cells were sorted. From this gate, CD8^+^/CD4^−^ cells were gated to determine CD8+ populations, and CD4^+^/Foxp3^+^ cells were gated to determine Treg populations. These gates are shown in the figure (Supplementary Fig. 7a, b). Similarly, for gating CD8^+^/Tetramer^+^ cells, CD45^+^/CD3^+^ cells were also first gated. From this gate, CD8^+^/Tetramer^+^ cells were gated as shown in the figure (Supplementary Fig. 7c, d). Similar gating schemes were used for gating splenic cells and dendritic cells as shown in other figure panels.

To assess ProIL2-, SumIL2-Fc-, or WTIL2-Fc-binding affinity, HEK-Blue^TM^ IL-2 cells (InvivoGen) were first stained with various dilutions of ProIL2, SumIL2-Fc, or WTIL2-Fc, then PE-conjugated donkey anti-human IgG was used as a secondary antibody. All staining steps were conducted at 4 °C in the dark. BD^TM^ Cytometric Bead Array (CBA) Mouse Inflammation Kit was used to measure the cytokines in the supernatants from mouse serum or tissue according to the manufacturer’s protocol (BD Biosciences). Data were collected on CytoFLEX flow cytometer (Beckman Coulter, Inc.) and analyzed by using CytExpert (Beckman Coulter, Inc.) or FlowJo (Tree Star Inc., Ashland, OR) software.

### Enzyme-linked immunosorbent assay (ELISA)

Microtiter plates (Corning Costar) were coated with 2 μg/mL (100 μL/well) capture antibody (AffiniPure Goat Anti-Human IgG, Fcγ fragment specific) overnight at 4 °C. After washing and blocking, diluted tissue lysate or serum from treated mice were added and incubated at 37 °C for 1 h. After washing, alkaline phosphatase (AP)-conjugated goat anti-human IgG (Fcγ fragment specific) was added and incubated at 37 °C for 30 min. Finally, the plates were visualized by adding 100 μL KPL Diethanolamine + pNPP substrate (Seracare Life Sciences) and read at 405 nm using the SPECTROstar Nano (BMG LABTECH).

For immunogenicity analysis, microtiter plates (Corning Costar) were coated with 2 μg/mL (100 μL/well) SumIL2-Fc, purified human IgG1 (Abbvie), or recombinant human IL-2 (Prometheus) overnight at 4 °C. After washing and blocking, purified mouse anti-human IgG or serum from treated mice were added and incubated at 37 °C for 1 h. After washing, peroxidase-conjugated goat anti-mouse IgG (Fcγ fragment specific) was added and incubated at 37 °C for 30 min. Finally, the plates were visualized by adding 100 μL 1-Step™ Ultra TMB-ELISA Substrate Solution (Thermo Fisher) and read at 650 nm using the SPECTROstar Nano (BMG LABTECH).

### Tumor growth and treatment

A total of 1 × 10^6^ MC38, 3 × 10^5^ B16, 3 × 10^5^ B16-OVA, 5 × 10^5^ CT26, or 5 × 10^5^ 4T1 cells were inoculated subcutaneously into right dorsal flanks of the mice in 100 μL PBS. Tumor-bearing mice were randomly grouped into treatment groups when tumors grew to around 50–100 mm^3^. For SumIL2-Fc treatment in MC38/B16/CT26 tumor-bearing mice, one dose of 1 mg/kg (300 pmol or 20 μg) was intraperitoneally given on day 9 (and day 12 for toxicity analysis). For ProIL2 treatment in MC38/B16/CT26 tumor-bearing mice, one dose of 1.5 mg/kg (300 pmol or 30 μg) was intraperitoneally given on day 9 (and day 12 for toxicity analysis). For ProIL2 treatment in 4T1 tumor-bearing mice, two doses of 1.5 mg/kg (300 pmol or 30 μg) was intraperitoneally given on days 9 and 12. For surgical treatment, tumors and surrounding skin were removed on day 14. For FTY720 treatment, 10 μg FTY720 was intraperitoneally administrated 1 day before ProIL2 treatment initiation and then 10 μg every other day for 2 weeks or until experiment termination; 200 μg anti-PDL1 was administered concurrently with ProIL2 treatment. For rechallenge experiments, 2.5 × 10^6^ 4T1 cells were inoculated subcutaneously into left dorsal flanks of mice in 100 μL PBS. Tumor volumes were measured by the length (*a*), width (*b*), and height (*h*) and calculated as tumor volume = *abh*/2.

### Production of fusion proteins

Based on the heterodimeric Fc variant KiHss-AkKh platform, SumIL2 was fused with knob variant Fc region, and the IL2Rβ/MMP linker was fused with hole variant Fc region. Relevant oligonucleotides used to synthesize these plasmids are shown in Supplementary Table 2. ProIL2 and SumIL2-Fc were generated by transient co-transfection of two arms of plasmids into FreeStyle^TM^ 293-F cells. IL-2 mutations in plasmids were determined and incorporated based on previous publications related to IL2Rβ-preferential IL-2 muteins^13,15^. The supernatant containing fusion proteins was purified using Protein A affinity chromatography according to the manufacturer’s protocol. The heterogeneity and purity were confirmed by SDS-PAGE.

### Tissue homogenate preparation

Spleen, lung, liver, and tumor were excised 1 day after treatment and homogenized in the Cell Lysis Kit (Bio-Rad Laboratories) with the FastPrep-24 5G Homogenizer. The samples were centrifuged for 10 min at 13,000*g*, and the supernatant was collected.

### Tumor digestion

Tumor tissues were excised and digested with 1 mg/mL Collagenase I (Sigma) and 0.5 mg/mL DNase I (Roche) at 37 °C for 30 min. The tumor cells were then passed through a 70 μm cell strainer to remove large pieces of undigested tumor. Tumor-infiltrating cells were washed twice with PBS containing 2 mM EDTA.

### Toxicity studies

Tumor-bearing mice were treated with two 300 pmol doses of treatment on days 9 and 12 after tumor inoculation. Tissues were extracted 2 days after the second treatment. Lung weights were measured, and then incubated at 60 °C for 48 h. Subsequent dry lung weight was measured, and the difference between initial and final lung weights was used to determine pulmonary wet weight. Livers were fixed in 10% neutral buffered formalin for 7 days before being embedded, sectioned, and processed for H&E staining. Serum was also collected 2 days after the second treatment for AST and ALT quantification.

### IL-2 reporter cell culture assay

Various dilutions of IL-2 fusion protein variants were incubated with 5 × 10^4^ HEK-Blue^TM^ IL-2 cells (InvivoGen) for 24 h as per manufacturer’s protocol. Afterwards, 25 μL cell supernatant was collected and incubated with 75 μL Quanti-Blue reagent (InvivoGen) for 1 h. The mixture was read at 650 nm using the SPECTROstar Nano (BMG LABTECH).

### MMP cleavage of ProIL2 and SDS-PAGE

Recombinant human MMP2 and MMP9 (BioLegend) were activated with 1 mM 4-Aminophenylmercuric acetate for 1 h as per manufacturer’s instructions. Recombinant human MMP14 (R&D Sciences) was activated with 0.1 μg/mL rhTrypsin (R&D Sciences) for 1 h and then incubated with 1 ng/μL AEBSF (R&D Sciences) for 15 min as per manufacturer’s instructions. Cleavage Assay Buffer consisted of 50 mM Tris, 10 mM CaCl_2_, 150 mM NaCl, and 0.05% (w/v) Brij 35 at pH 7.5. Either mock 4-APMA + rhTrypsin + AEBSF solution without MMPs, 50 ng MMP2, 50 ng MMP9, 100 ng MMP14, or the cocktail of all three MMPs were incubated with 1.5 μg ProIL2 in cleavage assay buffer at a total reaction volume of 10 μL at 37 °C for 1–4 h. Proteins were then run on SDS-PAGE and relative gel band intensities were analyzed to quantify the amount of cleavage in each ProIL2/MMP mixture. For an unprocessed example of this gel, please see the source data provided with this paper.

### Tissue homogenate immunoprecipitation and western blot assay

Tumor, liver, lung, and spleen tissues were homogenized as described above. Equivalent amounts of tissue homogenate by weight were incubated with Protein A-binding beads at a 100:1 ratio for 12 h at 4 °C. The beads were then isolated and run on a non-reducing SDS-PAGE. Proteins were transferred onto membrane using Trans-Blot Turbo manufacturer instructions. The membrane was incubated and rotated on shaker with 5% milk in PBS with 0.1% Tween 20 detergent for 1 h. After washing, horseradish peroxidase (HRP)-conjugated goat anti-human IgG (H + L) was added at a 1:2000 dilution and rotated on shaker for 1 h. The membrane was developed using SuperSignal West Dura Extended Duration Substrate and imaged with ChemiDoc Touch Imaging System (BioRad). For an unprocessed example of this western blot, please see the source data provided with this paper.

### IFN-γ enzyme-linked immunosorbent spot assay (ELISPOT)

B16-OVA (3 × 10^5^) tumors were injected subcutaneously on the right flank of C57BL/6 mice. PBS or 300 pmol of ProIL2 or SumIL2-Fc was intraperitoneally given on day 9. At 5 days after treatment, tumor-draining lymph nodes were collected, and 1–5 × 10^5^ lymph node cells were incubated with 5 μg/mL SIINFEKL peptide (OVA_257–264_) (or no peptide as a control) to stimulate the tumor-specific T cells. After 48 h of culture, ELISPOT assay was performed using the IFN-γ ELISPOT kit (BD Bioscience) according to the manufacturer’s instructions. IFN-γ spots were enumerated with the CTL-ImmunoSpot^®^ S6 Analyzer (Cellular Technology Limited).

### 4T1 metastatic tumor clonogenic assay

Lung tissues from 4T1 tumor-bearing or tumor-resected mice were excised and digested with 1 mg/mL Collagenase I (Sigma) and 0.5 mg/mL DNase I (Roche) at 37 °C for 30 min, and then passed through a 70 μm cell strainer to remove large pieces of undigested lung. Lungs cells were resuspended in 3 mL Dulbecco’s modified Eagle’s medium supplemented with 10% heat-inactivated fetal bovine serum, 100 U/mL penicillin, 100 U/mL streptomycin, and 10 μg/mL 6-thioguanine. The cells were plated in 6-well plates at a 1:400 dilution. After 6 days, cells were stained with crystal violet staining solution and colonies were counted.

### Statistical and power analysis

All the data analyses were performed with GraphPad Prism statistical software and shown as mean ± s.e.m. *p* value was determined by two-way ANOVA for tumor growth, log-rank test for survival, and Mann-Whitney non-parametric test for cytokine and ALT/AST studies (due to non-normal distributions). All tests were two-tailed without adjustment for multiple comparisons. Both unpaired *t*-tests and two- or multiple-way ANOVA were used for other analysis, with *t*-tests and two-way ANOVA yielding comparable significance results. Sample size was calculated using values of α = 0.05 and β = 0.20^55^. Effect sizes were determined using pilot studies of tumor growth curves and body weight: across multiple studies, standardized effect size averaged out to around 1.8. This resulted in a minimum sample size of 5 for each group, and our statistical analyses included at least 5 mice per group. A value of *p* < 0.05 was considered statistically significant.

### Reproducibility and variability

In each set of data, at least 2 experiments were performed. For half-life and tumor growth curves, each data point represents the aggregate of all replicates, and each replicate represents the data from one mouse. For column graphs, each data point represents one replicate, and each replicate represents the data from one mouse/tumor/tissue. In general, the variability of each experiment shown demonstrates the variability within each experiment. Occasionally, the variability between experiments is a little higher although the trends between experiments is the same (i.e., comparing Fig. 5e and Supplementary Fig. 7l).

### Reporting summary

Further information on research design and methods are available in the Nature Research Reporting Summary linked to this article.

## Supplementary information

Supplementary Information

Reporting Summary

## Data Availability

Source data are available as a Source Data file. The remaining data are available within the Article, Supplementary information, or available from the authors upon request.

## Source data

Source Data
