# Supplementary material for: A cytokine receptor-masked IL2 prodrug selectively activates tumor-infiltrating lymphocytes for potent antitumor therapy
Source: Nat Commun. 2021 May 13;12:2768. doi: 10.1038/s41467-021-22980-w (PMC8119481; doi:10.1038/s41467-021-22980-w)
Supplement: Supplementary file 1 — Supplementary Information [file 41467_2021_22980_MOESM1_ESM.pdf]

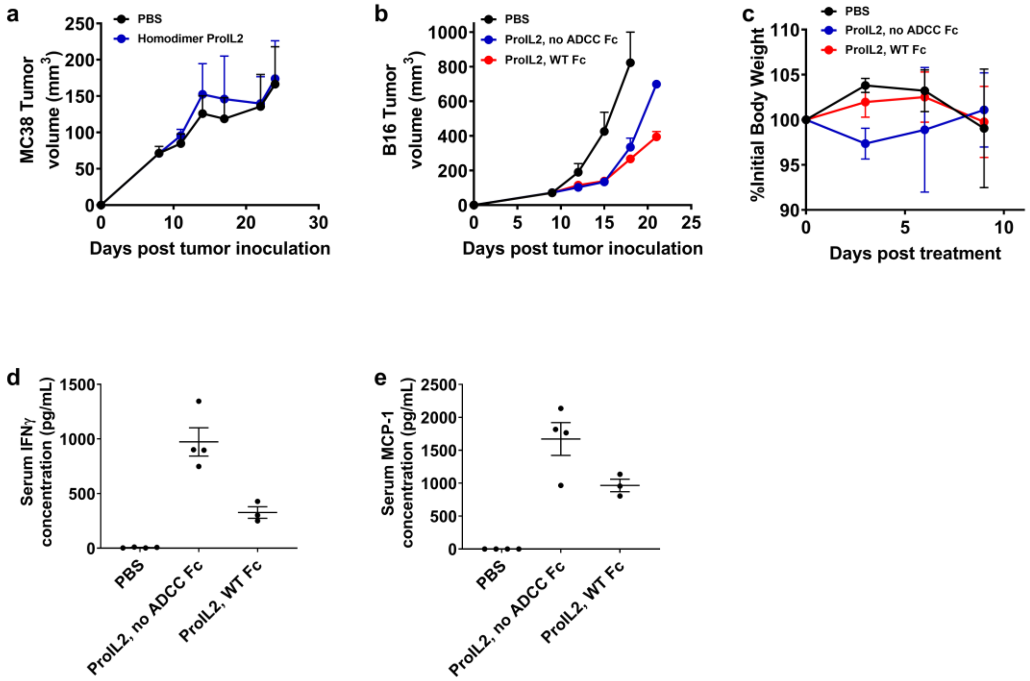

Supplementary Figure 1. Testing and optimizing ProIL2 designs. a MC38 s.c. tumor bearing mice were injected i.p. with PBS or a homodimeric form of ProIL2 (300 pmol) one time on day 9 post tumor inoculation; tumor growth was measured (n=1 experiment, total 8 individual mice for PBS treatment group, 4 for ProIL2). **b-e** B16 s.c. tumor bearing mice were injected i.p. with PBS, the best ProIL2 candidate with a no ADCC mutant Fc, or the best ProIL2 candidate with a WT hIgG1 Fc (300 pmol) one time on day 9 post tumor inoculation; **b-c** tumor growth and body weight were measured (n=1 experiment, total 4 individual mice per group). **d-e** Serum was collected and isolated from mice 24 hours post treatment, and Cytometric Bead Array was used to quantify the amount of serum IFNγ or MCP-1 (n=1 experiment, total 4 individual mice for PBS and ProIL2/no ADCC Fc, 3 for ProIL2/WT Fc). Data represent the mean ± s.e.m of replicates.

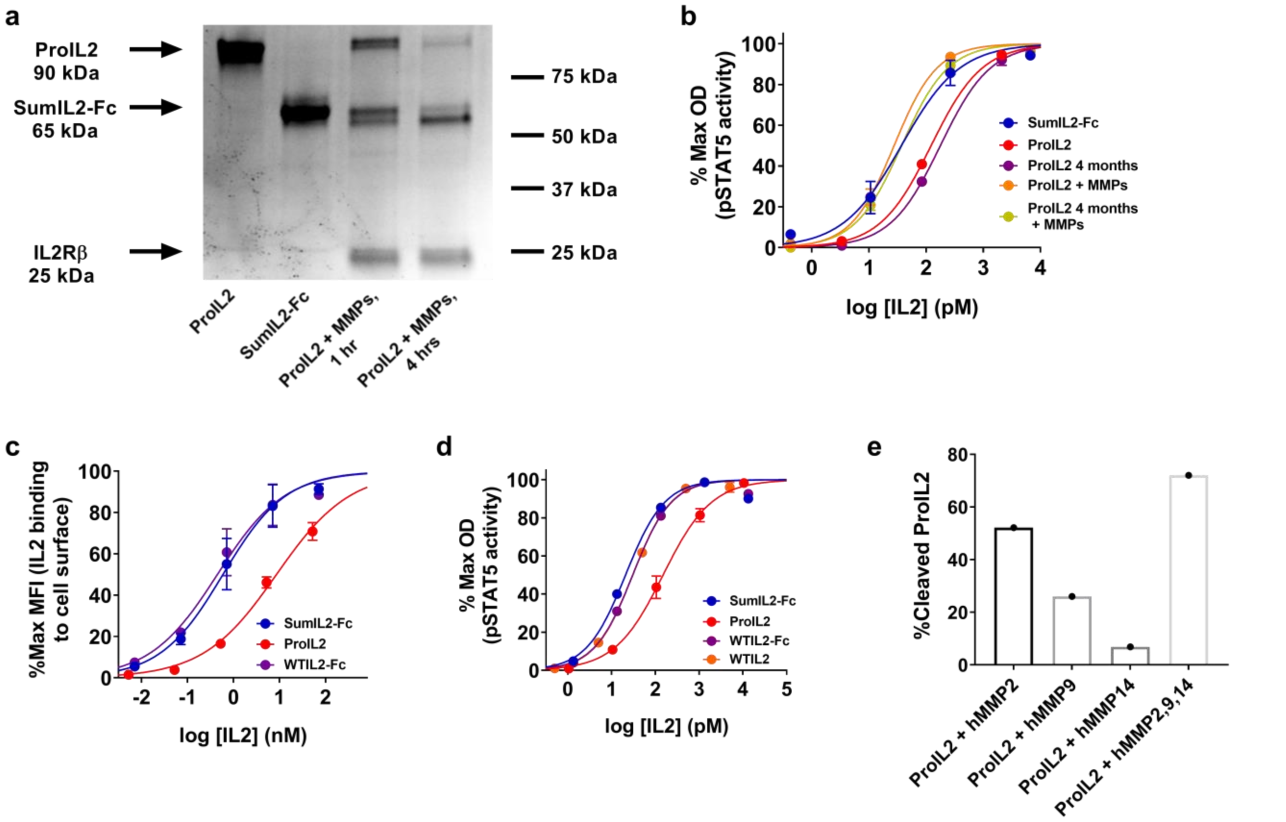

Supplementary Figure 2. Cleavage of ProIL2 *in vitro*. a ProIL2 was incubated with human MMP2, MMP9, and MMP14 for 1 or 4 hours, then run on non-reducing SDS-PAGE. ProIL2 and SumIL2-Fc were also run as controls. Other SDS-PAGE cleavage experiments (3 times) showed similar relative band intensities. **b** Functional activity of SumIL2-Fc, ProIL2 and ProIL2 that had been in 4°C for 4 months +/- incubation with human MMP2, MMP9, and MMP14 for 4 hours was assessed by using HEK-Blue^{TM} IL-2 Reporter cell assay (n=1 experiment, total 3 replicates per group). **c** Binding of SumIL2-Fc, ProIL2, and WTIL2-Fc at different doses to HEK-Blue^{TM} IL-2 Reporter cells was assessed via flow cytometry (n=2 experiments, total 2 replicates per group). **d** Functional activity of SumIL2-Fc, ProIL2, WTIL2-Fc, and recombinant WTIL2 was assessed by using HEK-Blue^{TM} IL-2 Reporter cell assay (n=1 experiment, total 3 replicates per group). **e** Protease activation of ProIL2. ProIL2 was incubated with human MMP2, MMP9, MMP14, or all 3 MMPs for 4 hours, then run on non-reducing SDS-PAGE, where gel band intensity was subsequently quantified (n=1 replicate per group). Data represent the mean ± s.e.m of replicates.

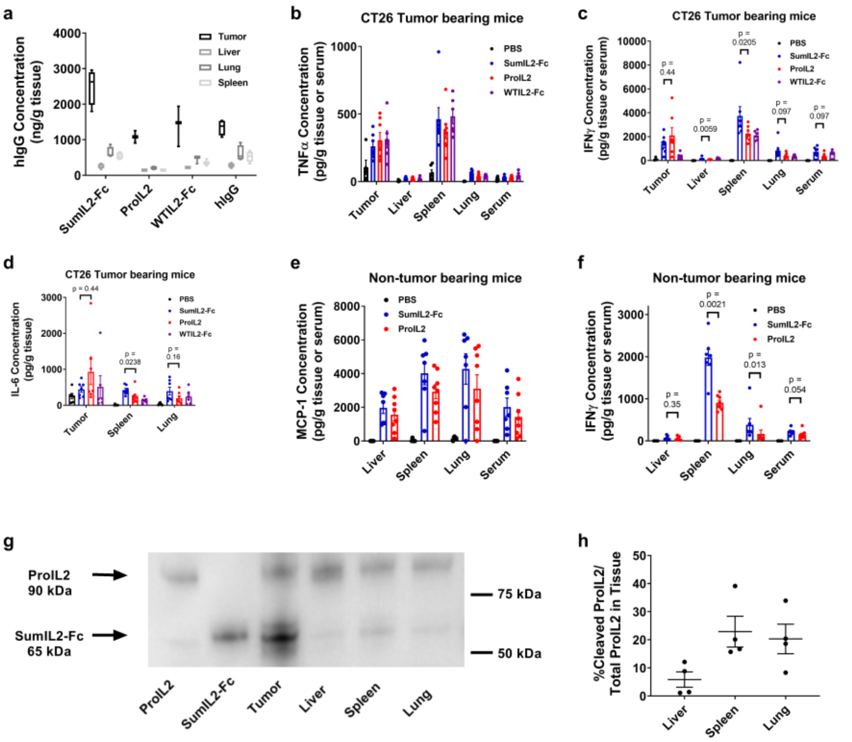

Supplementary Figure 3. Cleavage and biodistribution of ProIL2 *in vivo.* a-b PBS, 15 μg SumIL2-Fc, 20 μg ProIL2, 15 μg WTIL2-Fc, or 10 μg hIgG1 was injected i.p. into CT26 s.c. tumor bearing mice, and the labeled tissues were collected and homogenized 24 hours after treatment. **a** hIgG ELISA was used to quantify the amount ProIL2 in each homogenate, normalized by total tissue weight. Box and whisker plots with maxima, 75th percentile, center, 25th percentile, and minima are displayed (n=1 experiment, total 4 individual mice for SumIL2-Fc and hIgG treatment groups, 2 for ProIL2, 3 for WTIL2-Fc). **b** Cytometric Bead Array was used to quantify the amount of TNFα in either tissue homogenate or serum (n=2 experiments, total 5 individual mice for PBS treatment group, 7 for SumIL2-Fc and ProIL2, 6 for WTIL2-Fc). **c** PBS, 15 μg SumIL2-Fc, or 20 μg ProIL2 was injected i.p. into non-tumor bearing BALB/c mice, and the labeled tissues were collected and homogenized 24 hours after treatment. Cytometric Bead Array was used to quantify the amount of TNFα in each tissue homogenate or serum (n=2 experiments, total 5 individual mice for PBS treatment group, 7 for SumIL2-Fc, 8 for ProIL2). **d** 20 μg ProIL2 was injected i.p. into CT26 s.c. tumor bearing mice, and the labeled tissues were collected and homogenized 24 hours after treatment. hIgG immunoprecipitation of equivalent weights of tissue homogenate was performed with Protein A binding beads, and then run on Western Blot with hIgG binding antibody. Other Western Blots (from 7 other mice) showed similar relative band intensities. **e** 20 μg ProIL2 was injected i.p. into non-tumor bearing BALB/c mice, and the labeled tissues were collected and homogenized 24 hours after treatment. hIgG immunoprecipitation of equivalent weights of tissue homogenate was performed with Protein A binding beads, and then run on Western Blot with hIgG binding antibody (n=1 experiment, total 4 individual mice per group). Relative gel band intensity per lane was quantified. Data represent the mean ± s.e.m of replicates. Mann-Whitney U tests were performed to calculate p values.

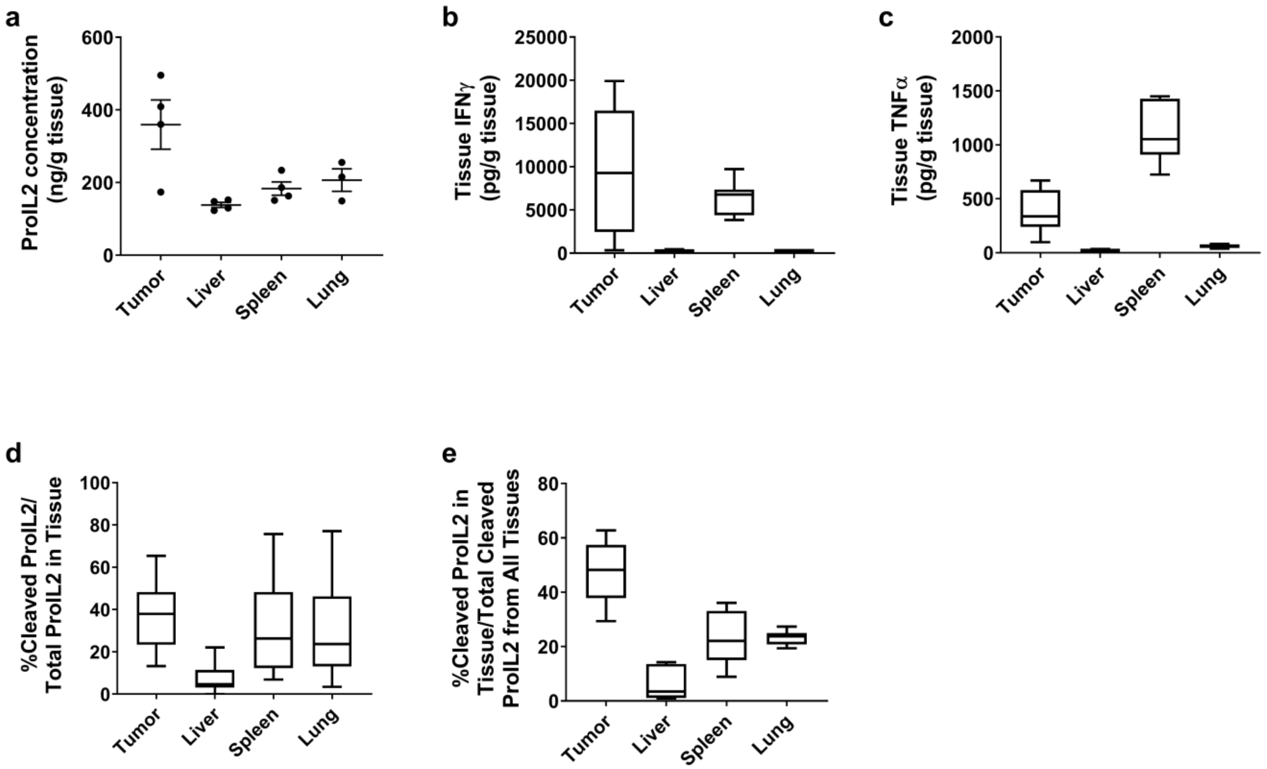

Supplementary Figure 4. Biodistribution of ProIL2 in B16 tumor bearing mice. 300 pmol ProIL2 was injected i.p. into B16 s. c. tumor bearing mice, and the labeled tissues were collected and homogenized 24 hours after treatment. **a** hIgG ELISA was used to quantify the amount ProIL2 in each homogenate, normalized by total tissue weight. (n=1 representative experiment of 2, total 3 individual mice per lung group, 4 for others). **b-c** Cytometric Bead Array was used to quantify the amount of IFNγ or TNFα in each tissue homogenate (n=2 experiments, total 8 individual mice per group). **d-e** hIgG immunoprecipitation of equivalent weights of tissue homogenate was performed with Protein A binding beads, and then run on Western Blot with hIgG binding antibody. **d** Relative gel band intensity per lane was quantified (n=4 experiments, total 13 individual mice per group). **e** Western Blot relative gel band intensities were quantified, and within each mouse, the total percentage of cleaved ProIL2 between each tissue was compared and quantified (n=2 experiments, total 6 individual mice per group). Box and whisker plots with maxima, 75th percentile, center, 25th percentile, and minima are displayed. Data represent the mean ± s.e.m of replicates.

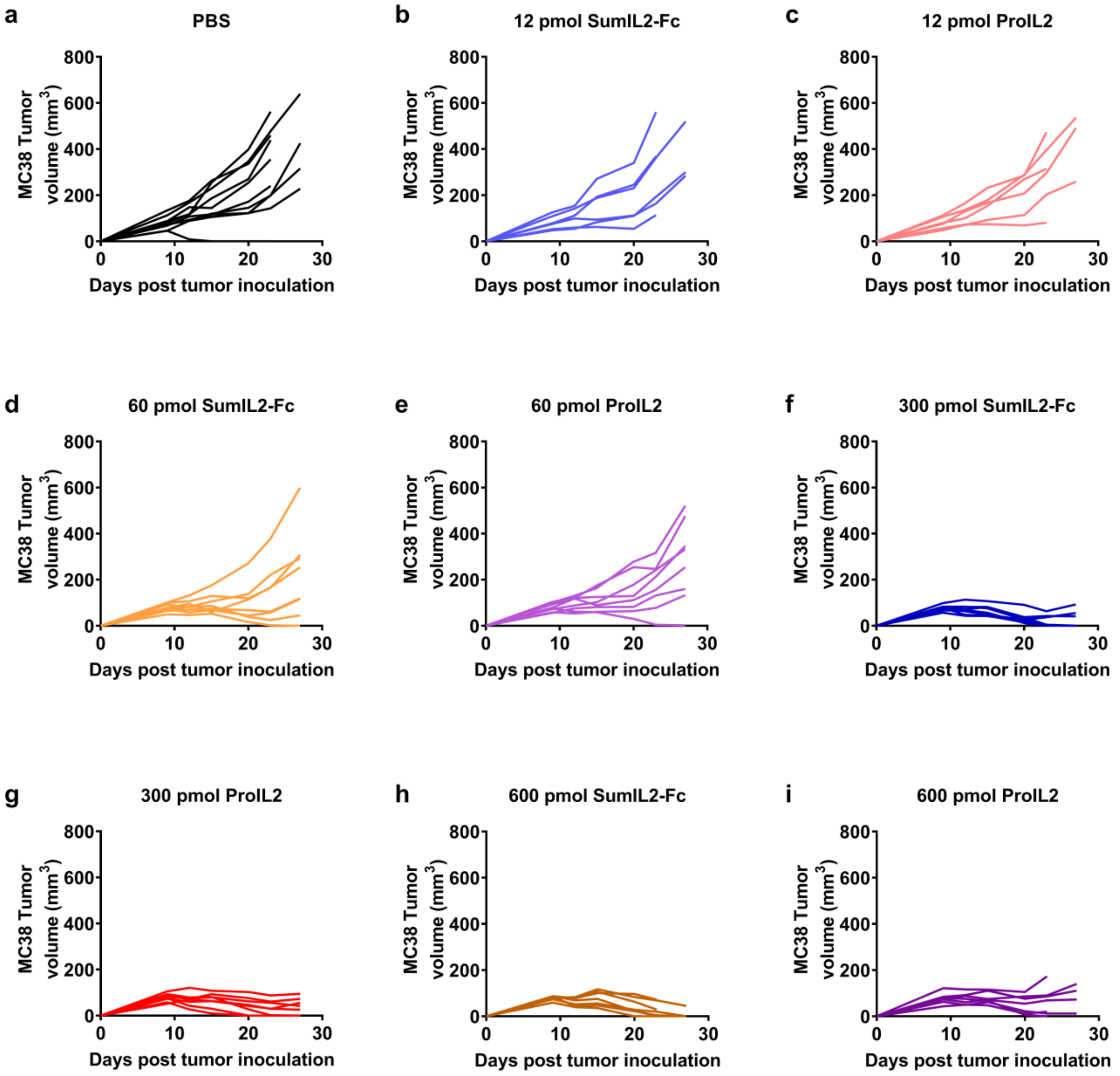

Supplementary Figure 5. Individual MC38 tumor growth curves for various doses of SumIL2-Fc and ProIL2. MC38 s.c. tumor bearing mice were injected i.p. with one dose of the labeled treatment on day 9 post tumor inoculation; tumor growth was measured. Each curve displays the MC38 tumor growth in one mouse (n=2 experiments, total 6 individual mice for 12 pmol treatment groups, 7 for 600 pmol treatment groups, 8 for 60 and 300 pmol treatment groups).

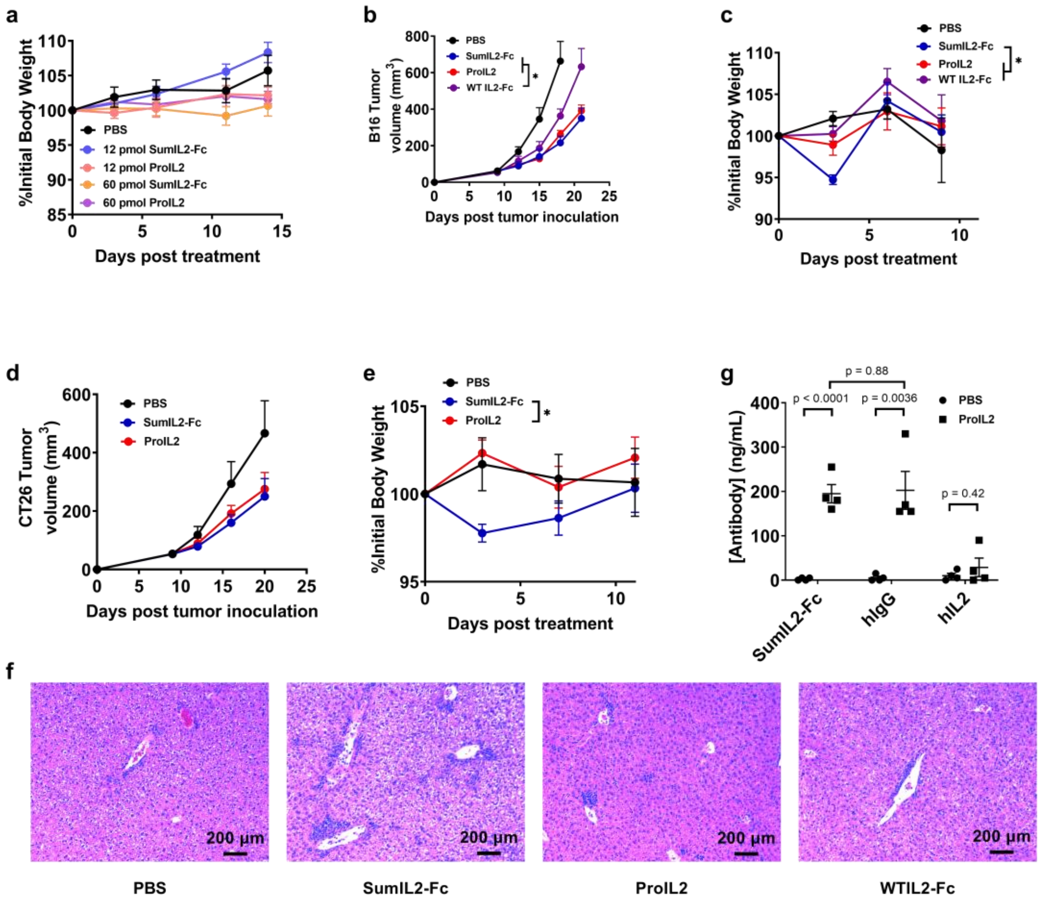

Supplementary Figure 6. ProIL2 has equivalent antitumor efficacy to SumIL2-Fc with reduced toxicity in multiple tumor models. a MC38 s.c. tumor bearing mice were injected i.p. with one dose of the labeled treatment on day 9 post tumor inoculation; body weight was measured (n=2 experiments, total 6 individual mice for 12 pmol treatment groups, 8 for PBS and 60 pmol treatment groups). **b-c** B16 s.c. tumor bearing mice were injected i.p. with PBS, SumIL2-Fc or ProIL2 (300 pmol) one time on day 9 post tumor inoculation; tumor growth ($p = 0.03$) and body weight ($p = 0.0086$) were measured (n=2 experiments, total 4 individual mice for WTIL2-Fc treatment group, 8 for others). **d-e** CT26 s.c. tumor bearing mice were injected i.p. with PBS, SumIL2-Fc or ProIL2 (300 pmol) one time on day 9 post tumor inoculation; tumor growth and body weight ($p = 0.0021$) were measured (n=1 experiment, total 4 individual mice per group). **f** B16 s.c. tumor bearing mice were injected i.p. with PBS, SumIL2-Fc, ProIL2, or WTIL2-Fc (150 pmol) two times on day 9 and day 12 post tumor inoculation. Livers were extracted and fixed in 10% formalin for 7 days, and then analyzed via H&E stain. Example 10x magnification liver staining from each treatment group is shown. Other liver samples (3 individual mice per treatment group) showed similar results. **g** B16 s.c. tumor bearing mice were injected i.p. with PBS or ProIL2 one time on day 9 post tumor inoculation. Serum was collected 6 days after treatment, and ELISA was used to quantify antibodies against SumIL2-Fc, hIgG, and human recombinant IL-2 as explained in the methods (n=1 experiment, 4 individual mice per group). Data represent the mean ± s.e.m of replicates. Student's t tests were performed to calculate p values.

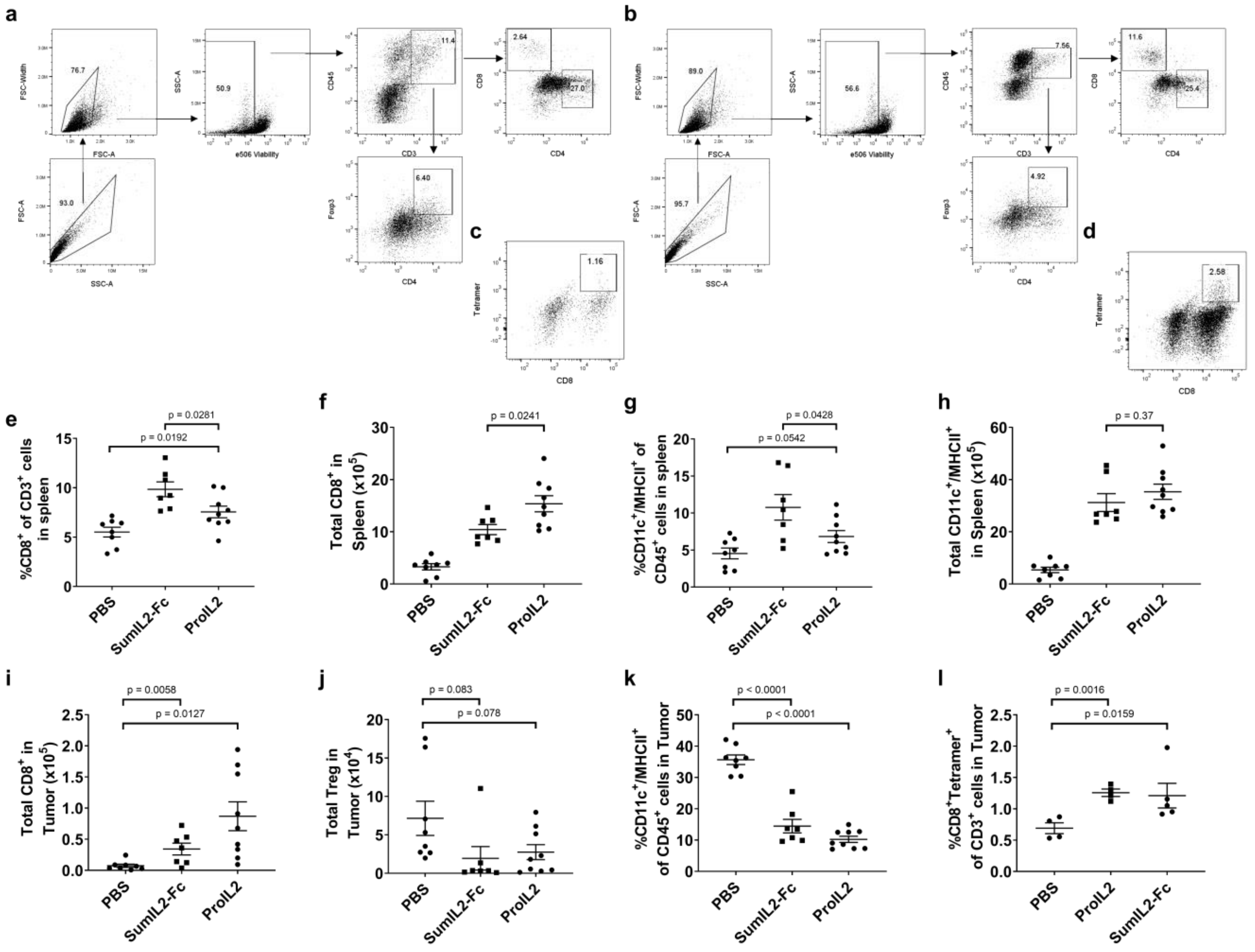

Supplementary Figure 7. ***In vivo*** **flow cytometry analysis of ProIL2.** B16 s.c. tumor bearing mice were injected i.p. with PBS, SumIL2-Fc or ProIL2 (300 pmol) two times on day 9 and day 12 post tumor inoculation. 48 hours after the last treatment, mice were euthanized and tumors were extracted, digested in collagenase/DNAse, and resuspended as single cells. Spleens from these mice were also extracted and resuspended as single cells. Representative flow cytometry gating strategies for CD8 T cells and Tregs from tumors from **a** PBS treated and **b** ProIL2 treated mice corresponding to Figures 5a-c are shown. The amounts of each labelled cell population from **e-g** spleens and **h-k** tumors in this figure were quantified (n=2 experiments, total 8 individual mice for PBS treatment group, 7 for SumIL2-Fc, 9 for ProIL2). **c-d** $CD8^+$ $Tetramer^+$ gating strategies from B16-OVA mice corresponding to Figure 5e are also shown. Data represent the mean ± s.e.m of replicates. Student's t tests were performed to calculate p values.

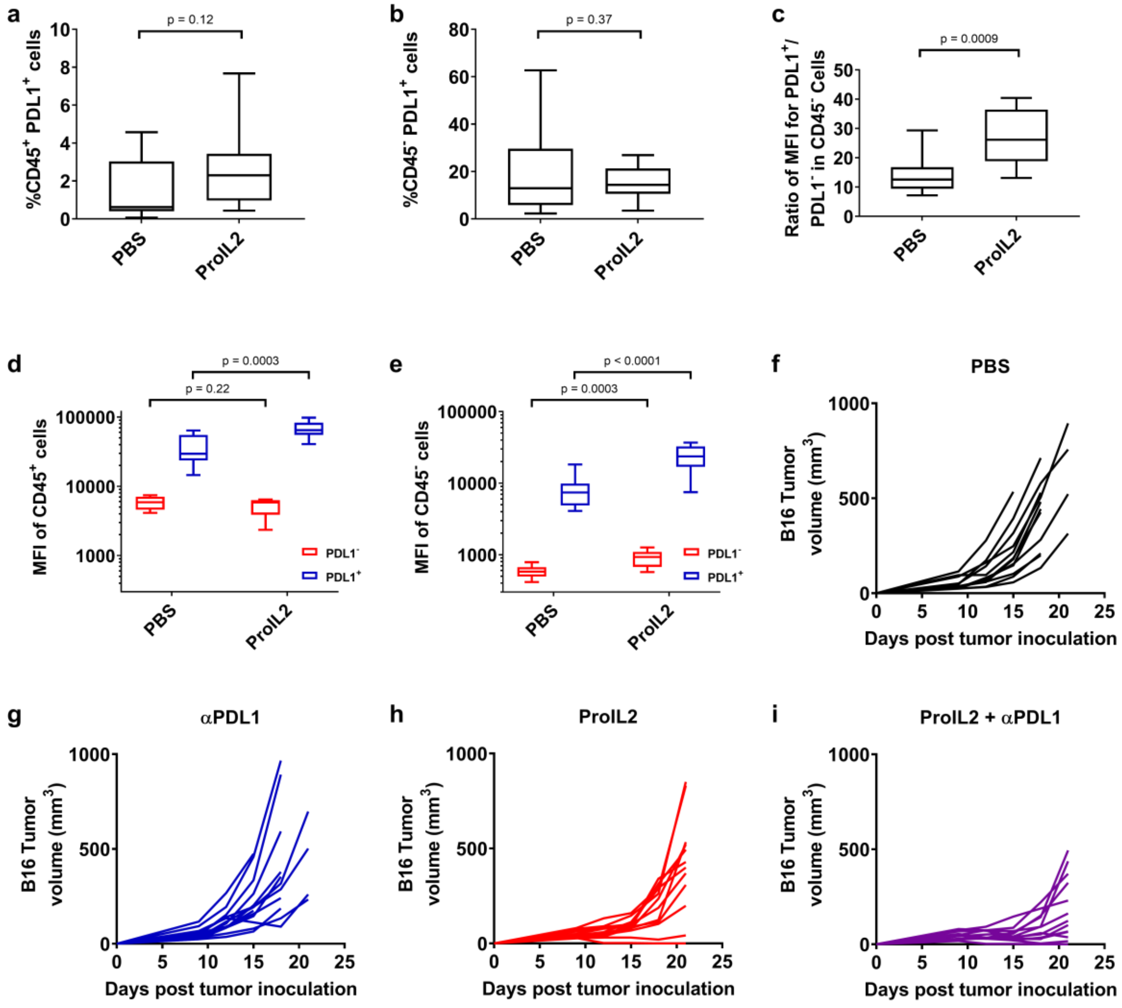

Supplementary Figure 8. ProIL2 can overcome tumor resistance to ICB. a-e B16 s.c. tumor bearing mice were injected i.p. with PBS or ProIL2 (300 pmol) one time on day 9 post tumor inoculation. 72 hours after treatment, mice were euthanized and tumors were extracted, digested in collagenase/DNAse, and resuspended as single cells. Box and whisker plots with maxima, 75th percentile, center, 25th percentile, and minima are displayed. **a-b** Flow cytometry was used to quantify the total percentage of $CD45^+/PDL1^+$ cells or $CD45^-/PDL1^+$ cells in each tumor. **c** In $CD45^-$ cells, the MFI of $PDL1^+$ and $PDL1^-$ cells were compared. **d-e** Raw MFI values of $CD45^+$ and $CD45^-$ cells are listed (n=3 experiments, total 12 individual mice per group). Data represent the mean ± s.e.m of replicates. Student's t tests were performed to calculate p values. **f-i** B16 s.c. tumor bearing mice were injected i.p. with PBS, anti-PDL1 (200 µg each), ProIL2 (300 pmol), or anti-PDL1 and ProIL2 one time on day 9 post tumor inoculation; tumor growth was measured and individual tumor growth curves are shown (n=3 experiments, total 12 individual mice for PBS and ProIL2 treatment groups, 13 for anti-PDL1 and combination groups).

Supplementary Table 1. ProIL2 full peptide sequence.

Sequences used to synthesize each domain of ProIL2 are shown.

**IL2RB**	AVNGTSQFTCFYNSRANISCVWSQDGALQDTSCQVHAWPDRRRWNQTCELLPVSQASWACNLILGAPDSQKLTTV<br>DIVTLRVLCREGVRWRVMAIQDFKPFENLRLMAPISLQVVHVETHRCNISWEISQASHYFERHLEFEARTLSPGHTWE<br>EAPLLTLKQKQEWICLETLTPDTQYEFQVRVKPLQGEFTTWSPWSQPLAFRTKPAALGKDT
**MMP Sequence**	SGARYRWLTA
	SGRSYAILTA
	SRSGRSPAIFTATG
	GSSGRSPAIFTAGS
	SGFIANPVTA
**Fc9**	DKTHTCPPCPAPELLGGPSVFLFPPKPKDTLMISRTPEVTCVVVDVSHEDPEVKFNWYVDGVEVHNAKTKPREEQY<br>NSTYRVVSVLTVLHQDWLNGKEYKCKVSNKALPAPIEKTISKAKGQPREPQVYTLPPCRDELTKNQVSLWCLVKGFY<br>PSDIAVEWESNGQPENNYKTTPPVLDSDGSFFLYSALTVDKSRWQQGNVFSCSVMHEALHNHYTQKSLSLSPG
**SumIL2**	APTSSSTKKTQLQLEHLLLDLQMILNGINNYKNPKLTRMLTAKFYMPKKATELKHLQCLEEELKPLEEVLNLAQSKNFH<br>FDPRDVVSNINVFVLELKGSETTFMCEYADETATIVEFLNRWITFCQSIISTLT
**Fc6**	DKTHTCPPCPAPELLGGPSVFLFPPKPKDTLMISRTPEVTCVVVDVSHEDPEVKFNWYVDGVEVHNAKTKPREEQY<br>NSTYRVVSVLTVLHQDWLNGKEYKCKVSNKALPAPIEKTISKAKGQPREPQVCTLPPSRDELTKNQVSLSCAVKGFY<br>PSDIAVEWESNGQPENNYKTTPPVLDSDGSFKLVSKLTVDKSRWQQGNVFSCSVMHEALHNHYTQKSLSLSPGK

Supplementary Table 2. Key Resources used.

Key reagents, assays, cell lines, organisms, and DNA are displayed.

REAGENT or RESOURCE	SOURCE	IDENTIFIER
Antibodies		
Anti-PDL1 Atezolizumab	Genentech	N/A
Human IgG1(kappa)	Abbvie	N/A
Anti-CD45 (Flow cytometry, 30-F11)	BioLegend	Cat# 103126
Anti-CD8 (Flow cytometry, 53-6.7)	BioLegend	Cat# 100730
Anti-CD3 (Flow cytometry, 145-2C11)	BD Biosciences	Cat# 564379
Anti-CD4 (Flow cytometry, RM4-5)	BD Biosciences	Cat# 550954
Anti-Foxp3 (Flow cytometry, MF-14)	BioLegend	Cat# 126408
Anti-PD-L1 (Flow cytometry, 10F.9G2)	BioLegend	Cat# 124308
Anti-CD11c (Flow cytometry, N418)	BioLegend	Cat# 117306
Anti-MHCII (Flow cytometry, M5.114.15.2)	eBioscience	Cat# 56-5321-82
Fixable Viability Dye eFluor™ 506	Thermo Fisher	Cat# 65-0866-18
Anti-FcγIII/II receptor (clone 2.4G2)	BD Biosciences	Cat# 553141
Goat Anti-Human IgG-HRP	Santa Cruz Biotech	Cat# sc-2453
iTAg Tetramer/PE - H-2 Kb OVA (SIINFEKL)	MBL	Cat# TB-5001-1
AffiniPure Goat Anti-Human IgG, Fcγ fragment specific	Jackson ImmunoResearch	Cat# 109-005-098
Alkaline Phosphatase-AffiniPure Goat Anti-Human IgG, Fcγ fragment specific	Jackson ImmunoResearch	Cat# 109-055-098
Peroxidase-AffiniPure Goat Anti-Mouse IgG, Fcγ fragment specific	Jackson ImmunoResearch	Cat# 115-035-071
Donkey Anti-Human IgG (H+L)	Jackson ImmunoResearch	Cat# 709-116-149
Purified Mouse Anti-Human IgG	BD Biosciences	Cat# 555784
Chemicals, Peptides, and Recombinant Proteins		
FTY720 (hydrochloride)	Selleckchem	Cat# S5002
Dulbecco's Modified Eagle's Medium	Sigma- Aldrich	Cat# D6429
Collagenase type I	Sigma	Cat# C0130
DNase I	Roche	Cat# 11284932001
OVA257-264 (SIINFEKL)	Invivogen	Cat# vac-sin
Recombinant human IL-2 (aldesleukin)	Prometheus	N/A
6-Thioguanine, 98%, Alfa Aesar	Thermo Fisher	Cat# AAB2128003
Recombinant Human MMP-2 (carrier-free)	BioLegend	Cat# 554302
Recombinant Human MMP-9 (carrier-free)	BioLegend	Cat# 550502
4-Aminophenylmercuric acetate	Sigma-Aldrich	Cat# A9563
Recombinant Human MMP-14/MT1-MMP (NS0-expressed) Protein, CF	R&D Systems	Cat# 918-MPN-010
Recombinant Human Active Trypsin 3/PRSS3 Protein, CF	R&D Systems	Cat# 3714-SE-010

AEBSF, CF	R&D Systems	Cat# EI001
QUANTI-Blue^{TM}	InvivoGen	Cat# rep-qbs
Experimental Models: Cell Lines		
B16	ATCC	Cat# CRL-6322
MC38	ATCC	N/A
CT26	ATCC	Cat# CRL-2638
4T1	ATCC	Cat# CRL-2539
FreeStyle^{TM} 293-F	Thermo Fisher	Cat# R79007
HEK-Blue^{TM} IL-2 Cells	InvivoGen	Cat# hkb-il2
Key Oligonucleotides/Primers		
IL2 Forward	ATTTCGCGAGCCCCTACAAGCAGCAGCACCA	
IL2 Reverse	ATTTTCGAAGGTCAGTGTGCTGATGATGCTCTGGC	
IL2RB Forward	ATTTCGCGAGCCGTGAATGGCACCA	
IL2RB Reverse	ATTTTCGAATGTGTCCTTGCCCA	
MMP Fc Forward Seq 1	ATTATTTCGAAGGTGGCGGTGGATCCTCTAGAAGCGGCAGGTCTCCTGCCATCTTTACAGCCGGTGGCGGTGGATCCGACAAAACTCAC	
MMP Fc Forward Seq 2	ATTTTCGAAGGTGGCGGTGGATCCAGCGGCGCCAGGTACAGGTGGCTGACCGCCGGCTCTAGCGGAGGATCCGACAAAACTCAC	
MMP Fc Forward Seq 3	ATTATTTCGAAGGTGGCGGTGGATCCTCTAGAAGCGGCAGGTCTCCTGCCATCTTTACAGCCACCGGTGGTGGCGGTGGATCCGACAAAACTCAC	
GS Fc Forward	ATTTTCGAAGGTGGCGGTGGATCCGGTGGCGGTGGATCCGGTGGCGGTGGATCCGGCTCTAGCGGAGGATCCGACAAAACTCAC	
Fc Reverse	ATGGCTGATTATGATCAATGAATT	
Recombinant DNA		
Plasmid: pEE6.4-IL2Rβ-MMP-Fc9		
Plasmid: pEE6.4-SumIL2-Fc6		
Plasmid: pEE6.4-Fc9		
Plasmid: pEE6.4-WTIL2-Fc6		
