# Supplementary material for: A cytokine receptor-masked IL2 prodrug selectively activates tumor-infiltrating lymphocytes for potent antitumor therapy
Source: Nat Commun. 2021 May 13;12:2768. doi: 10.1038/s41467-021-22980-w (PMC8119481; doi:10.1038/s41467-021-22980-w)
Supplement: Supplementary file 2 — Reporting Summary [file 41467_2021_22980_MOESM2_ESM.pdf]

nature research

Corresponding author(s): Xuezhi Cao, Yang-Xin Fu

Last updated by author(s): Mar 22, 2021

Reporting Summary

Nature Research wishes to improve the reproducibility of the work that we publish. This form provides structure for consistency and transparency in reporting. For further information on Nature Research policies, see our Editorial Policies and the Editorial Policy Checklist.

Statistics

For all statistical analyses, confirm that the following items are present in the figure legend, table legend, main text, or Methods section.

n/a	Confirmed	
☐	☒	The exact sample size (n) for each experimental group/condition, given as a discrete number and unit of measurement
☐	☒	A statement on whether measurements were taken from distinct samples or whether the same sample was measured repeatedly
☐	☒	The statistical test(s) used AND whether they are one- or two-sided<br>*Only common tests should be described solely by name; describe more complex techniques in the Methods section.*
☐	☒	A description of all covariates tested
☐	☒	A description of any assumptions or corrections, such as tests of normality and adjustment for multiple comparisons
☐	☒	A full description of the statistical parameters including central tendency (e.g. means) or other basic estimates (e.g. regression coefficient) AND variation (e.g. standard deviation) or associated estimates of uncertainty (e.g. confidence intervals)
☐	☒	For null hypothesis testing, the test statistic (e.g. F, t, r) with confidence intervals, effect sizes, degrees of freedom and P value noted<br>*Give P values as exact values whenever suitable.*
☒	☐	For Bayesian analysis, information on the choice of priors and Markov chain Monte Carlo settings
☒	☐	For hierarchical and complex designs, identification of the appropriate level for tests and full reporting of outcomes
☒	☐	Estimates of effect sizes (e.g. Cohen's d, Pearson's r), indicating how they were calculated

Our web collection on statistics for biologists contains articles on many of the points above.

Software and code

Policy information about availability of computer code

Data collection	Beckman Coulter CytExpert Software v2.3, Nanodrop, Bio-Rad Image Lab Software
Data analysis	GraphPad Prism software v9.0, Bio-Rad Image Lab Software v5.2.1, CTL-Immunospot S6 Analyzer v7.0, FlowJo v10

For manuscripts utilizing custom algorithms or software that are central to the research but not yet described in published literature, software must be made available to editors and reviewers. We strongly encourage code deposition in a community repository (e.g. GitHub). See the Nature Research guidelines for submitting code & software for further information.

Data

Policy information about availability of data

All manuscripts must include a data availability statement. This statement should provide the following information, where applicable:

- Accession codes, unique identifiers, or web links for publicly available datasets
- A list of figures that have associated raw data
- A description of any restrictions on data availability

The data that support the findings of this study are available from the corresponding author on reasonable request.

Field-specific reporting

Life sciences study design

All studies must disclose on these points even when the disclosure is negative.

Sample size	Sample sizes were determined from the similar experiments in the former publications of the group. Power analysis calculations were also performed as described in the statistical analysis section of the methods to determine the minimum number of samples necessary for 80% power.
Data exclusions	No data were excluded.
Replication	At least 2 independent experiments were performed for each figure panel. The performed replications were successful.
Randomization	Mice in this study were matched with age and tumor size, and were randomly allocated to the different groups.
Blinding	Collection of replicate animal samples was not blinded in order to properly collect paired tissue/serum at different time points. However, data collection (i.e. tumor growth, flow cytometry) and analysis were blinded.

Reporting for specific materials, systems and methods

We require information from authors about some types of materials, experimental systems and methods used in many studies. Here, indicate whether each material, system or method listed is relevant to your study. If you are not sure if a list item applies to your research, read the appropriate section before selecting a response.

Materials & experimental systems

n/a	Involved in the study	
☐	☒	Antibodies
☐	☒	Eukaryotic cell lines
☒	☐	Palaeontology and archaeology
☐	☒	Animals and other organisms
☒	☐	Human research participants
☒	☐	Clinical data
☒	☐	Dual use research of concern

Methods

n/a	Involved in the study	
☒	☐	ChIP-seq
☐	☒	Flow cytometry
☒	☐	MRI-based neuroimaging

Antibodies

Antibodies used	BD Biosciences anti-mouse FcγIII/II receptor (2.4G2), BioLegend anti-mouse CD45 (30-F11), BioLegend anti-mouse CD8 (53-6.7), BD Biosciences anti-mouse CD3 (145-2C11), BD Biosciences anti-mouse CD4 (RM4-5), Biolegend anti-mouse Foxp3 (MF-14), BioLegend anti-mouse PD-L1 (10F.9G2), Biolegend anti-mouse CD11c (N418), eBioscience anti-mouse MHCII (M5.114.15.2), Thermo Fisher Fixable Viability Dye eFluor 506, Santa Cruz Biotech Goat anti-human IgG-HRP, MBL iTAg Tetramer/PE - H-2 Kb OVA (SIINFEKL), Jackson ImmunoResearch AffiniPure Goat Anti-human IgG Fcγ fragment specific, Jackson ImmunoResearch Alkaline Phosphatase-AffiniPure Goat Anti-Human IgG Fcγ fragment specific, Jackson ImmunoResearch Peroxidase-AffiniPure Goat Anti-Mouse IgG Fcγ fragment specific, Jackson ImmunoResearch Donkey Anti-Human IgG (H+L), BD Biosciences Purified Mouse Anti-Human IgG, Genentech anti-PDL1 Atezolizumab, Abbvie Human IgG1(kappa)
Validation	Antibody validations (i.e. knockout and knockdown testing, flow cytometry application, etc.) were performed by suppliers and have been published by our group and others. Please see Biolegend, BD Biosciences, and Jackson ImmunoResearch manufacturer websites for validation data (i.e. https://www.biolegend.com/en-us/kokd-validation).

Eukaryotic cell lines

Policy information about cell lines

Cell line source(s)	ATCC (B16, MC38, CT26, 4T1)
Authentication	The cell lines obtained from ATCC with responsive authentication and characterization. Morphology, karyotyping, and PCR analysis were used to authenticate each cell line.
Mycoplasma contamination	All the cell lines used in this study was tested negative for mycoplasma contamination.
Commonly misidentified lines (See ICLAC register)	None

Animals and other organisms

Policy information about studies involving animals; ARRIVE guidelines recommended for reporting animal research

Laboratory animals	8 week old female C57BL/6J and BALB/c mice were purchased from The Jackson Laboratory. All mice were maintained under specific pathogen-free conditions at the University of Texas Southwestern Medical Center. Animal care and experiments were carried out under institutional and National Institutes of Health protocol and guidelines.
Wild animals	The study did not involve wild animals.
Field-collected samples	The study did not involve field-collected samples
Ethics oversight	This study has been approved by the Institutional Animal Care and Use Committee of the University of Texas Southwestern Medical Center.

Note that full information on the approval of the study protocol must also be provided in the manuscript.

Flow Cytometry

Plots

Confirm that:

- [x] The axis labels state the marker and fluorochrome used (e.g. CD4-FITC).
- [x] The axis scales are clearly visible. Include numbers along axes only for bottom left plot of group (a 'group' is an analysis of identical markers).
- [x] All plots are contour plots with outliers or pseudocolor plots.
- [x] A numerical value for number of cells or percentage (with statistics) is provided.

Methodology

Sample preparation	Tumor tissues were excised and digested with 1 mg/mL Collagenase I (Sigma) and 0.5 mg/mL DNase I (Roche) in the 37°C for 30mins, tumor was then passed through a 70 µm cell strainer to remove large pieces of undigested tumor. Tumor infiltrating cells were washed twice with PBS containing 2 mM EDTA.
Instrument	Beckman Coulter CytoFlex
Software	Beckman Coulter CytExpert
Cell population abundance	Physical parameter and Thermo Fisher Fixable Viability Dye was used to exclude dead cells. Positive populations were defined using not stained cells as reference. Isotype controls were used to confirm the specificity of the staining.
Gating strategy	Positive populations were defined using not stained cells as reference. Isotype controls were used to confirm the specificity of the staining.

- [x] Tick this box to confirm that a figure exemplifying the gating strategy is provided in the Supplementary Information.
